# Molecular and expression analysis indicate the role of CBL interacting protein kinases (CIPKs) in abiotic stress signaling and development in chickpea

**DOI:** 10.1038/s41598-022-20750-2

**Published:** 2022-10-07

**Authors:** Nikita Poddar, Deepika Deepika, Pragya Chitkara, Amarjeet Singh, Shailesh Kumar

**Affiliations:** 1 Bioinformatics Lab, National Institute of Plant Genome Research (NIPGR), Aruna Asaf Ali Marg, New Delhi, 110067 India; 2 Stress Signalling Lab, National Institute of Plant Genome Research (NIPGR), Aruna Asaf Ali Marg, New Delhi, 110067 India

**Keywords:** Computational biology and bioinformatics, Plant stress responses

## Abstract

Calcineurin B-like proteins (CBL)-interacting protein kinases (CIPKs) regulate the developmental processes, hormone signal transduction and stress responses in plants. Although the genome sequence of chickpea is available, information related to the *CIPK* gene family is missing in this important crop plant. Here, a total of 22 *CIPK* genes were identified and characterized in chickpea. We found a high degree of structural and evolutionary conservation in the chickpea CIPK family. Our analysis showed that chickpea *CIPK*s have evolved with dicots such as *Arabidopsis* and soybean, and extensive gene duplication events have played an important role in the evolution and expansion of the *CIPK* gene family in chickpea. The three-dimensional structure of chickpea CIPKs was described by protein homology modelling. Most CIPK proteins are localized in the cytoplasm and nucleus, as predicted by subcellular localization analysis. Promoter analysis revealed various *cis*-regulatory elements related to plant development, hormone signaling, and abiotic stresses. RNA-seq expression analysis indicated that *CIPK*s are significantly expressed through a spectrum of developmental stages, tissue/organs that hinted at their important role in plant development. The qRT-PCR analysis revealed that several *CaCIPK* genes had specific and overlapping expressions in different abiotic stresses like drought, salt, and ABA, suggesting the important role of this gene family in abiotic stress signaling in chickpea. Thus, this study provides an avenue for detailed functional characterization of the *CIPK* gene family in chickpea and other legume crops.

## Introduction

South Asia is the major producer of chickpea, the world’s second most important food legume crop. India is the largest producer of chickpeas in South Asia, and is credited for about 70% of the world’s chickpeas production; thereby contributing an estimated production of 5.9 million tonnes (mt) annually^1^. Chickpea is an important dietary source for vegetarians due to the presence of vital nutritive constituents in the seeds, including 20–30% crude proteins, 40% carbohydrates, and 3–6% fat^2^. In addition to this, chickpea seeds are rich in minerals, such as calcium, magnesium, potassium, phosphorus, iron, and zinc^3^. Chickpea production is severely affected by various stresses, which results in a huge gap between its demand and supply. Abiotic stresses alone account for an estimated 40–60% of global chickpea production losses annually. Drought causes major damage, and accounts for about 50% of chickpea yield loss. Temperature fluctuations and soil salinity combined are responsible for about 25% yield loss of chickpea^4^. The chickpea yield loss due to drought, cold, and salinity, respectively cost approximately 1.3 billion, 186 million, and 354 million US dollars, which has economically dented several chickpea‐producing countries^5^. These stresses harm flower set, pollen viability, pod set/abortion, and retention. As all these crucial developmental stages essentially determine seed number, a negative impact on these stages significantly hampers the chickpea yield. Due to continuous climate changes, severe and frequent challenges of drought are predicted in arid and semi-arid areas, where chickpea is traditionally cultivated^6^ and can be detrimental to overall crop productivity. Thus, identification and utilization of important stress-related genes in biotechnological programmes to generate improved chickpea varieties is the need of the hour.

Environmental cues, such as biotic and abiotic stresses are known to elicit the increase in cytosolic Ca^2+^ with specific spatiotemporal features. The accumulation of Ca^2+^ generates a specific “Ca^2+^ signature” in the form of spikes, waves, and oscillations. The stimulus-specific Ca^2+^ signature is decoded by Ca^2+^ sensors and downstream effectors generating a response^7^. Several Ca^2+^ sensors have been identified, and characterized in plants, including calmodulin (CaM) and CaM-like proteins (CMLs)^8^, Ca^2+^-dependent protein kinases (CDPKs)^9^ and calcineurin B-like proteins (CBLs)^1^. Among these, CBLs are a unique group of Ca^2+^ sensors and to determine their functional identity, a family of plant-specific serine/threonine kinases; CBL-interacting protein kinases (CIPKs) function as important downstream signaling components^10^. In higher plants, both CBLs and CIPKs are encoded as multi-gene families. A total of 10 CBL and 26 CIPK members have been identified in *Arabidopsis*, whereas 10 CBLs and 33 CIPKs are identified in rice^11^. A large number of CBL and CIPK members constitute a complex, and sophisticated signaling network. For instance, in *Arabidopsis*, each CBL interacts with multiple CIPKs, and vice-versa^12^, consequently, some CBLs share a common CIPK partner, and some CIPKs are regulated by a common CBL protein. Such specific and overlapping patterns of CBL-CIPK interactions may provide functional specificity and synergism to CBL-CIPK signaling networks.

As far as structure is concerned, CBLs are typical Ca^2+^ sensor proteins with four EF-hand domains, which are responsible for Ca^2+^ binding. On the other hand, CIPKs harbor several functionally distinct domains. CIPKs consist of a conserved catalytic kinase domain at the N-terminal, and a regulatory domain at the C-terminal^10,12^. Typical of a functional kinase protein, the CIPK kinase domain contains an ATP binding site and an activation loop. The regulatory domain contains FISL/NAF, and PPI motifs which are responsible for the interaction of CIPK with CBL and type 2C protein phosphatases, respectively^13,14^. The function of CBL-CIPK pathways is found to be regulated by the pattern of gene expression, Ca^2+^ binding affinity, protein stability, and protein–protein interactions^7^. CBL-CIPK networks have been implicated in diverse functions that regulate plant response to biotic stress^15–17^, abiotic stress^1,18,19^, nutrient deficiency^1,20,21^, metal toxicity^22,23^, and plant development^24–27^. The majority of knowledge about CBL-CIPK signaling has been obtained from the research in the model plant *Arabidopsis thaliana*. Detailed information about CBL-CIPK networks, and their function is scarce in the legume crop chickpea. Although the CBL family has been identified in chickpea^28^, the identification and characterization of the *CIPK* gene family is still missing. A comprehensive gene expression profiling of the *CIPK* gene family will help to understand the CBL-CIPK functions in chickpea. Information obtained from expression analysis will encourage the utilization of crucial genes for genetic engineering of chickpea plants towards better stress tolerance and development.

With this rationale, we have identified and characterized the *CIPK* gene family in chickpea. Phylogenetic analysis and chromosomal localization studies have provided insight into the evolution and expansion of this gene family in chickpea. Analysis of gene and domain structures ensured the authenticity and integrity of identified genes. Homology modelling helped to understand the three-dimensional structure of chickpea CIPKs. In-silico analysis revealed the *cis*-regulatory elements in *CaCIPK* gene promoters responsible for different stresses, hormones and development. In chickpea, gene expression profiles identified by using various publicly available datasets, suggest the involvement of the *CaCIPK* family in abiotic stress signaling, and seed development. Experimental validation by qRT-PCR indicated the role of this gene family in abiotic stress signaling in chickpea.

## Material and methods

### Identification of CIPKs in the chickpea genome

The chickpea genome submitted by (Varshney et al. 2013) was downloaded from NCBI and explored for the identification of *CIPK* encoding genes. Rice and *Arabidopsis thaliana* CIPK proteins were retrieved from Uniprot (Swiss-Prot), and a homology search was performed using BLAST tool (E-value = 10^−6^) against the chickpea proteome. Significant hits were selected based on >  = 50% identity, and >  = 100 amino acid length alignment. Further, the HMM sequence of the CIPK-NAF domain was extracted from Pfam (http://pfam.xfam.org/) database, and a BLAST search was done (E-value = 10) against the chickpea proteome. Furthermore, both sets of putative candidates were mixed, and redundant sequences were removed using CD-HIT tool^29^. The domain analysis was performed using the standalone version of InterproScan^30^. Gene attributes such as gene ID, protein ID, CDS, size of amino acid, and chromosomal coordinates were extracted from the NCBI web server.

### Phylogenetic analysis

To examine the evolutionary relationship between *CIPK*s in chickpea and other species, Multiple Sequence Alignment (MSA) was performed with the amino acid sequences of CIPKs from four different plant species e.g. *Arabidopsis thaliana*, *Oryza sativa*, *Glycine max*, and *Cicer arietinum*) using ClustalW^31^ at default settings in MEGA X version 10.1.8^32^. The neighbor-joining method was used to construct the phylogenetic tree and bootstrap values were calculated with 1000 replicates to determine the phylogenetic relationship among the CIPKs. The iTOL^33^ web-server was used to mark the different clades of CIPKs with different colors, and shapes for better visualization.

### Gene structures, motif organization, and domain prediction

Gene Structure Display Server (GSDS)^34^ was used to compare the CDS sequences of *CIPK* genes with their corresponding genomic DNA sequences to investigate the coding sequences and intron structure. Motif organization of CIPK proteins was examined via the Multiple Expression motifs for Motif Elicitation (MEME) tool^35^ with default parameters; site distribution- zero or one occurrence per sequence; motif discovery mode-classic; motif length 6–50; and the top ten most enriched motifs were selected based on lowest E-values. The identification of domains was performed by standalone package of InterProScan^30^. The coordinates of essential domains and active sites were extracted and further used as an input in the Illustrator for Biological Sequences^36^ for visualization.

### Gene nomenclature, chromosomal location, and gene duplication

Nomenclature of *CIPK* genes in chickpea was based on the sequence and phylogenetic analysis. Identified genes were named as *CaCIPK* followed by a number corresponding to their respective *Arabidopsis* orthologs. Details of various features, such as locus ID, CDS, protein size, isoelectric point (pI), no. of introns were obtained from NCBI and ExPASy. Gene localization was displayed on different chromosomes by using TBtools^37^. To search the duplicated gene pairs within the chickpea genome, the protein sequence of chickpea CaCIPKs was used to run the all-versus-all local BLASTP with parameters of E-value 1e− 5, max target sequences 5 and m6 format output. The MCScanX software package^38^ was used to analyse the segmentally duplicated regions of *CIPK* genes in chickpea. Genes and the intra-species collinear gene pairs were mapped to the eight chromosomes of chickpea using the family_circle_plotter.java script. The protein sequences of each duplicate gene pair were aligned by CLUSTALW. The alignment file in FASTA format and the CDS sequences of the corresponding genes were used to calculate the non-synonymous (Ka) and synonymous (Ks) substitution values by the PAL2NAL server^39^.

### In silico promoter analysis

For the identification of *cis*-regulatory elements in the promoters of chickpea *CIPK* genes, 2000 bp upstream sequences of the coding region of genes were extracted from NCBI, and used as input in the PlantCARE (http://bioinformatics.psb.ugent.be/webtools/plantcare/html/) tool.

### Subcellular localization and physicochemical properties of CaCIPK proteins

The full-length CIPK protein sequences of chickpea were used as input to predict their subcellular localization using the CELLO program^40^. The locations were displayed in different parts of the cell by Biorender software (https://biorender.com/). The online tool Compute pI/MW of Expasy^41^ was used to calculate the molecular weight (MW) and isoelectric point (pI) of CaCIPKs.

### Protein–protein interaction network construction for CaCBLs-CaCIPKs

To elucidate the interaction network between CIPK and CBL proteins in chickpea, the amino acid sequences of 22 CIPKs*,* and 9 CBLs from the study of^28^ were used as an input for STRING (http://string-db.org/) tool. In the STRING tool, the interaction network can be constructed using a low confidence value of 0.15, medium confidence of 0.4, high confidence of 0.7, and highest confidence of 0.9^42^. Experimental data of CBL and CIPK interaction in chickpea was constructed by using the confidence value > 0.4. Homologous proteins of the chickpea in the network were identified by reciprocal best BLASTP analysis from chickpea proteins.

### Protein tertiary structure prediction

The tertiary structures of the 22 CaCIPK proteins were predicted by Phyre2 web portal (http://www.sbg.bio.ic.ac.uk/phyre2). It uses advanced remote homology detection methods to build 3D models for protein sequences^43^. All the proteins were modelled with 100% confidence by the single highest scoring template model.

### Expression analysis using RNA-seq data

To generate the genome-wide expression profiles of *CaCIPK* genes in different tissues and developmental stages, we investigated the available RNA-Seq data extracted from Sequence Read Archive (SRA; SRP121085) of NCBI. Expression data was obtained for 27 tissues belonging to different developmental stages namely, germinating stage (embryo, plumule, and radical), seedling stage (epicotyl and primary root), vegetative stage (stem, root, leaf, and, petiole), reproductive stage (bud, flower, immature seed, pod, stem, leaf, petiole, nodules, and, root) and senescence stage (petiole, leaf, stem, leaf yellow, mature seed, immature seed, root nodule, and seed coat). For different seed development stages in two distinct desi chickpea varieties (JGK3 and Himchana 1), RNA-seq data was extracted from SRA accession numbers SRP072563 and SRP072564. The expression was analysed for seven seed stages representing early-embryogenesis (S1), mid-embryogenesis (S2), late-embryogenesis (S3), mid-maturation (S5), and late-maturation (S7) seeds.

In order to better understand the expression profiles of CIPK genes, we performed a comparative analysis of the same in the closely related legume e.g. *Cajanus cajan,* at different developmental stages, and two important abiotic stresses viz. salt and heat stress. The RNA-seq data, from the BioProjects PRJNA344973 and PRJNA354681, for 33 distinct tissues representing five different developmental stages, namely, Reproductive stage (embryo sac, seed, pod wall, mature seed, immature seed, mature pod, shoot apical meristem, sepal, petal, petiole, stem, immature pod, nodule, root, pistil, stamen, flower, bud, and leaf), seedling (root and shoot), senescence stage (root, leaf, petiole, and stem), vegetative stage (leaf, root, nodule, and shoot apical meristem), and germinal stage (embryo, hypocotyl, radical, and cotyledon) were used. The gene expression data from the BioProjects PRJNA382795 and PRJNA343064, of the salt-tolerant (ICP7) and salt susceptible (ICP1071) genotypes belonging to two different tissues e.g. root and shoot, under salt stress conditions were analysed. While the RNA-seq data for studying heat stress was retrieved from BioProject PRJNA635234, where *Cajanus* leaves were subjected to stress for 30 min and three hours respectively.

Raw RNA-Seq reads downloaded from SRA were processed by using FASTP^44^, to remove the adapter, poly-N, short, and low-quality reads. The reference genome of chickpea was downloaded from the NCBI genome web server. HISAT2^45^ tool was used to build the index of the reference genome, and for the mapping of filtered reads onto the genome index. The alignments were assembled into potential transcripts using StringTie^46^, and the transcript abundance was calculated as fragments per kilobase of transcript per million reads (FPKM) values. For the differential expression analysis, three biological replicates of each treatment and control were analyzed, and fold change expression was calculated by the ratio of average FPKM of test samples, and average FPKM of control samples. The ‘pheatmap’ package of R was used to generate the heatmaps of the expression data using the logarithm of normalized expression values for the tissue study, and the logarithm of fold change for the remaining studies.

### Plant growth and stress treatment

For gene expression analysis, Desi chickpea (var. ICC4958) was used. Seeds were surface sterilized and the plants were cultivated according to previous study^47^. Different stress treatments were applied to 10-day-old seedlings. Water was withheld and seedlings were air dried within the folds of tissue paper at 22–23 °C temperature to simulate drought stress. After 0 h, 1 h, 3 h, and 6 h, of drought treatment, samples were collected in triplicate. Seedlings were housed in a 150 mM NaCl solution in a beaker for salt stress, and samples were taken at 0 h, 3 h, 6 h, and 12 h. Seedlings were kept in 100 μM ( ±) ABA in sterile water in a beaker under light for ABA treatment, and samples were taken after 0 h, 3 h, 6 h, and 12 h. Untreated seedlings (of the same age) from respective time points were used as controls for different abiotic stress treatments.

### RNA extraction and cDNA synthesis

The TRIzol reagent (Ambion, life technologies) was used to extract RNA from 100 mg tissue of the control, drought, salinity, and ABA treated root and shoot samples, in accordance with manufacturer's protocol. Using the RNeasy Min Elute Clean-up Kit (QIAGEN), the RNA was purified to remove any genomic DNA contamination. The ratio 1.8–2.0 for A260:A280 and 2.0–2.3 for A260:A230 was used to determine the quantity and quality of RNA using Nano Drop ONEc (Thermo Scientific) nano-spectrophotometer. The integrity of the RNA was then confirmed using MOPS-agarose gel electrophoresis. The RevertAid first strand cDNA synthesis kit (Thermo Scientific) was used to create first strand cDNA from 1 μg total RNA, in accordance with the manufacturer's protocol.

### Expression analysis by qRT-PCR

As mentioned in previously^48^, PRIMER EXPRESS SOFTWARE was used to design qRT-PCR primers for specific genes. After real-time PCR run, their specificity was determined using the RGAP BLAST program and melt curve analysis. Table S1 lists all of the primers in detail. The expression pattern was assessed using three biological replicate samples (with three technical replicates of each biological replicate) of control and nutrient-deficient roots and shoots. In accordance to previous study^49^, the gene expression was detected by Bio-rad CFX96 real-time PCR system (Bio-Rad) using iTaq Universal SYBR Green supermix (Bio-Rad).

### Statistical analysis

All expression and quantitative assays were replicated three times in order to perform statistical analysis. The data is presented as the average of three replicates ± SD (standard deviation). The statistical significance among the replicate samples was determined using a two-tailed student's t-test. *p*-value < 0.05 or 0.01 or 0.005 were regarded as statistically significant, and marked by *, ** and *** respectively.

## Results

### Identification and sequence analysis of *CaCIPK* genes

In chickpea, a total of 39 putative CIPK protein sequences were obtained by the homology search with *Arabidopsis* and rice CIPKs. Further HMM profile search against chickpea proteome revealed 38 putative CIPK sequences. After combining both sets, followed by manual curations, a total of 26 unique CIPK sequences were obtained. Domain analysis revealed that the necessary domains like PPI and NAF domain, were absent in four sequences, therefore, they were removed from the list. Finally, a total of 22 non-redundant *CIPK* encoding genes were fetched from chickpea genome.

The length of 22 CaCIPK proteins varied from 418 aa (CaCIPK16) to 503 aa (CaCIPK12) with an average molecular weight of 51.16 kDa. Most of the CaCIPK proteins (except CaCIPK3 and CaCIPK11) were found to have an isoelectric point (pI) greater than 7 (Table 1). To gain insights into the homology of the CaCIPK proteins, the sequence identity and similarity were calculated using the SIAS tool (http://imed.med.ucm.es/Tools/sias.html). This analysis showed that the CaCIPKs have 46.13 to 81.87% sequence similarity among themselves. Four protein pairs viz. CaCIPK15/18, CaCIPK5/7, CaCIPK1/17 and CaCIPK2/10 showed a high degree of identity i.e., 76.67%, 76.39%, 76.01% and 73.84%, respectively (Figure S1). Even the most divergent protein pair of CaCIPK8 and CaCIPK22 shared 35.49% identity (49.18% similarity).

**Table 1:** Summary of various features of the chickpea CIPK family.

Transcript								Protein			
Gene name	NCBI ID	Chromosome	Start	End	NCBI identifier	Introns	CDS length	NCBI identifier	Length (aa)	Isoelectric point (pI)	Protein wt. (kDa)
*CaCIPK1*	LOC101499928	Ca1	25,702,213	25,709,285	XM_004488130.3	11	1347	XP_004488187.1	448	8.02	50.34
*CaCIPK2*	LOC101488582	Ca2	27,180,279	27,182,763	XM_004490648.3	0	1368	XP_004490705.1	455	9	51.9
*CaCIPK3*	LOC101491417	Ca2	33,011,673	33,018,054	XM_004491146.3	13	1326	XP_004491203.1	441	6.59	50.44
*CaCIPK4*	LOC101504371	Ca4	32,563,304	32,564,889	XM_004497948.3	0	1305	XP_004498005.1	434	9.12	49.29
*CaCIPK5*	LOC101513526	Ca6	53,224,442	53,226,557	XM_012717669.2	0	1353	XP_012573123.1	450	8.26	51.83
*CaCIPK6*	LOC101511702	Ca7	1,293,373	1,294,939	NM_001309656.1	0	1335	NP_001296585.1	444	9.11	50.34
*CaCIPK7*	LOC101496895	Ca1	8,839,486	8,841,422	XM_004486556.3	0	1347	XP_004486613.1	448	9.14	50.77
*CaCIPK8*	LOC101511605	Ca2	23,829,223	23,836,498	XM_027331998.1	14	1425	XP_027187799.1	474	8.03	53.78
*CaCIPK9*	LOC101493574	Ca1	36,737,555	36,744,930	XM_027334254.1	14	1398	XP_027190055.1	465	8.93	52.14
*CaCIPK10*	LOC101498077	Ca5	26,642,943	26,645,602	XM_004500288.3	0	1392	XP_004500345.1	463	8.76	52.43
*CaCIPK11*	LOC101499591	Ca5	26,665,837	26,667,956	XM_012715825.2	0	1320	XP_012571279.1	439	6.49	48.88
*CaCIPK12*	LOC101506657	Ca1	3,314,537	3,316,964	XM_012712534.2	0	1512	XP_012567988.1	503	7.14	56.66
*CaCIPK13*	LOC101489428	Ca5	28,720,785	28,723,545	XM_004500487.3	0	1380	XP_004500544.1	459	9.04	51.78
*CaCIPK14*	LOC101488926	Ca2	27,205,251	27,207,496	XM_004490649.3	1	1308	XP_004490706.1	435	8.67	48.82
*CaCIPK15*	LOC101507945	Ca6	37,592,424	37,596,217	XM_004506380.3	0	1395	XP_004506437.1	464	8.82	52.36
*CaCIPK16*	LOC101510050	Ca4	44,315,144	44,317,032	XM_004498761.3	0	1257	XP_004498818.1	418	8.94	47.54
*CaCIPK17*	LOC101510187	Ca7	3,876,530	3,882,455	XM_012717926.2	11	1344	XP_012573380.1	446	8.43	50.8
*CaCIPK18*	LOC101492060	Ca3	21,042,046	21,046,759	XM_004492487.3	0	1392	XP_004492544.1	463	8.41	52.63
*CaCIPK19*	LOC101507330	Ca7	6,850,555	6,859,747	XM_012718074.2	13	1341	XP_012573528.1	446	9.2	50.84
*CaCIPK20*	LOC101506991	Ca1	3,307,254	3,309,153	XM_012712535.2	0	1362	XP_012567989.1	453	8.96	52.14
*CaCIPK21*	LOC101512343	Ca7	2,026,868	2,030,503	XM_012717974.2	13	1359	XP_012573428.1	452	8.17	51.07
*CaCIPK22*	LOC101493164	Ca3	21,099,833	21,101,565	XM_004492489.3	0	1296	XP_004492546.1	431	8.89	48.89

### Gene and domain structure

**Figure 1:**
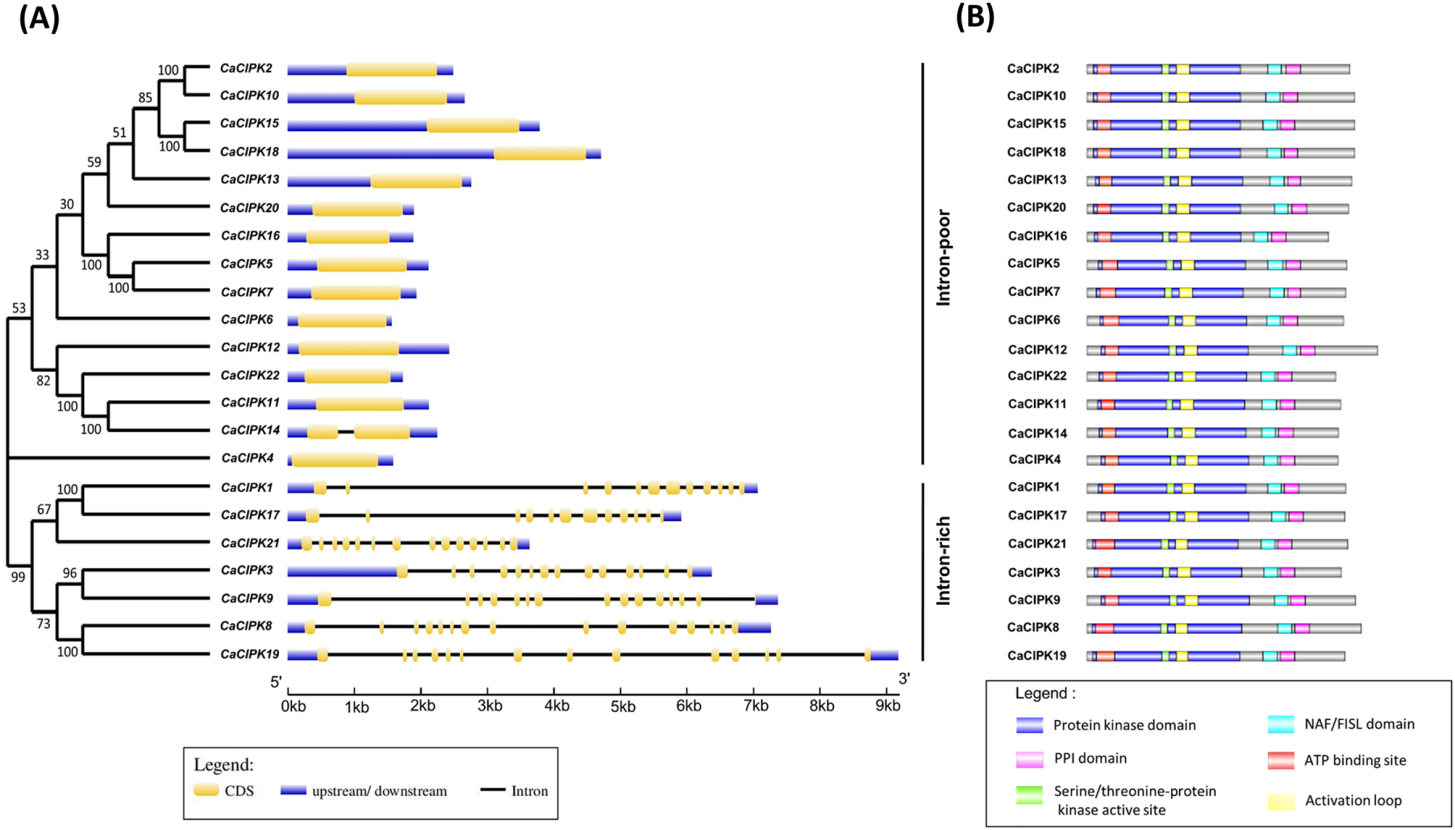
Structural features of chickpea CIPK family. (**A**) Exon–intron organization is shown for 22 *CaCIPK* genes. The gene structure was drawn using GSDS program. The yellow boxes represent exons, the lines represent introns and the blue boxes represent upstream/downstream UTRs. The values in the phylogenetic tree (left side) represent bootstrap values. (**B**) Protein structure of chickpea CIPK family showing conserved protein kinase, NAF and PPI domain along with some important sites present at the N-terminal e.g. ATP binding site, Serine/threonine-protein kinase active site and activation loop.

The evolution of gene families is often reflected by their gene structure^50,51^. There is a large variation in the number of introns in *CIPK* genes in chickpea, which ranges from 0 to 14 (Fig. 1A). Out of 22 *CaCIPK* genes, only seven have more than two introns. Thus, *CaCIPKs* could be classified into two groups: (1) intron-poor subgroup with zero (*CaCIPK2, -4, -5, -6, -7, -10, -11, -12, -13, -15, -16, -18, -20, -22*) or one (*CaCIPK14*) intron, and (2) intron-rich subgroup with greater than 10 introns (*CaCIPK1, -3, -8, -9, -17, -19, -21*).

The domain structure analysis revealed that all the CaCIPK proteins possess three essential domains; a N-terminal kinase domain, and at C-terminal, a regulatory NAF domain and protein phosphatase interaction (PPI) domain (Fig. 1B). Multiple sequence alignment of 22 CaCIPK proteins showed that the glycine residue of ‘DFG’ in the activation loop was changed to asparagine (DFN) in CaCIPK1, whereas the alanine of ‘APE’ was modified to serine (SPE) in CaCIPK6 and CaCIPK18 (Figure S2). Important sites for phosphoregulation of activation loop i.e., serine, threonine, and tyrosine^52^, were found to be conserved in all CaCIPK proteins except CaCIPK6, where serine was modified to cysteine. In CaCIPK proteins, a total of 10 conserved motifs were identified by using the MEME tool. Out of those, motif 7 was annotated as NAF domain due to the presence of conserved asparagine-alanine-phenylalanine residues^13^, whereas motif 8, which is located just after motif 7, was designated as the PPI domain as it contains important arginine and phenylalanine residues. All motifs, except motif 6 and 10, were present in 22 CaCIPK proteins (Figure S3). Motif 6 was absent in CaCIPK4, while motif 10 was absent in the subgroup of intron-rich CaCIPKs. The sequence logo of different motifs is depicted in Table S2.

### Phylogenetic analysis of CaCIPK family

**Figure 2:**
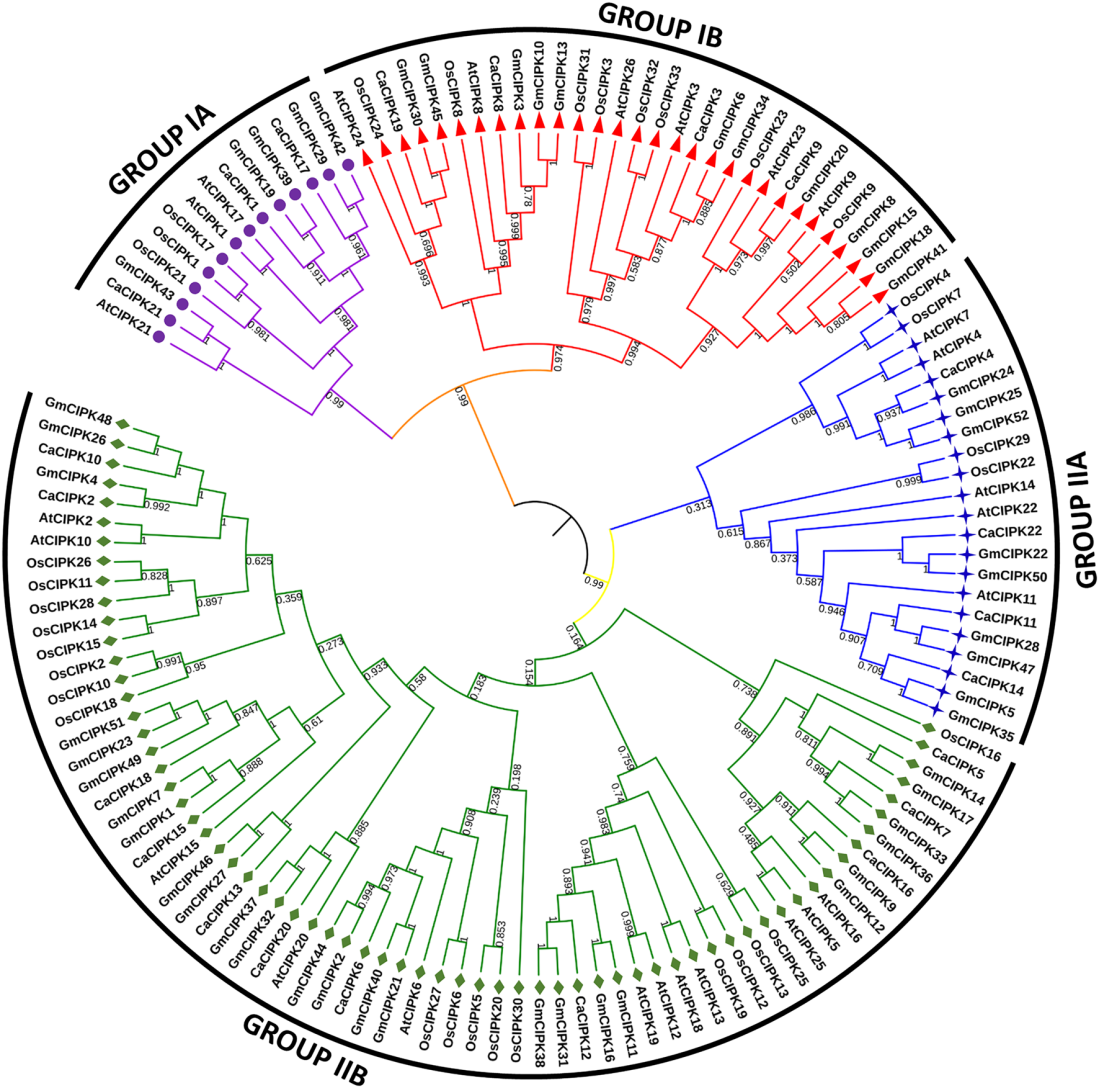
Phylogenetic relationship among CIPKs from different plants. Evolutionary relationship from *Arabidopsis thaliana* (At), *Oryza sativa* (Os), *Glycine max* (Gm) and *Cicer arietinum* (Ca) constructed using Neighbor-Joining method by using MEGA X software is shown via phylogenetic tree. Labels above the nodes represent bootstrap values calculated from 1000 replicates. The Groups IA, IB, IIA and IIB are indicated by violet, red, blue and green branch lines respectively.

A total of 133 CIPK protein sequences from four species viz. *Arabidopsis thaliana* (26), *Oryza sativa* (33), *Glycine max* (52), and *Cicer arietinum* (22) were used to construct the phylogenetic tree to explore the evolutionary relationship among the CIPKs. Based on high bootstrap values, the tree was divided into two major groups e.g. group I and II. These two groups were further sub-divided into IA, IB, and IIA, IIB (Fig. 2). Group IA includes CaCIPK1, *-*17, *-*21, group IB includes CaCIPK3, *-*8, *-*9 and *-*19, Group IIA includes CaCIPK4, *-*11, *-*14 and *-*22 and Group IIB includes CaCIPK2, *-*5, *-*6, *-*7, *-*10, *-*12, *-*13, *-*15, *-*16, *-*18 and *-*20. Group IIB contained most members of CaCIPKs.

### Chromosomal location and gene duplication of *CaCIPK* genes

**Figure 3:**
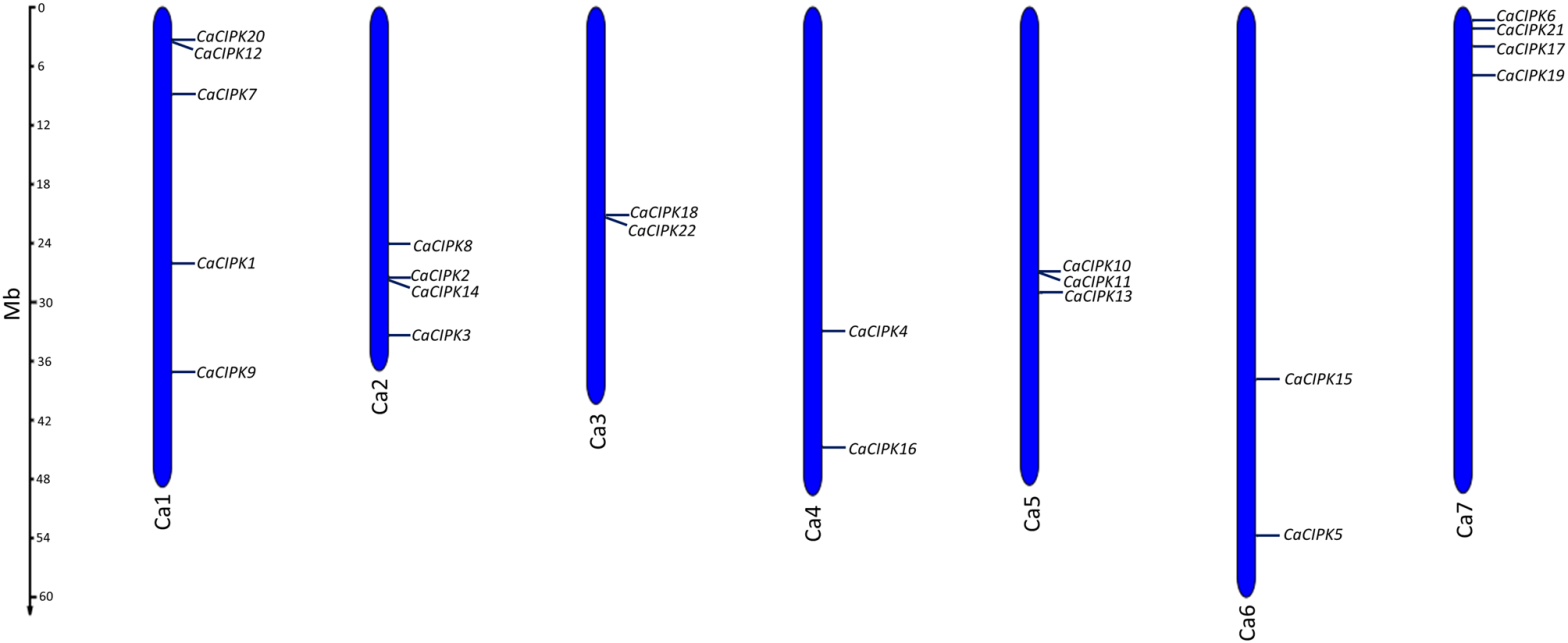
Chromosomal locations of chickpea *CIPK* genes. Blue colour bars represent the chromosomes, the location of genes has been marked alongside. Chromosome numbers are given at the bottom of each chromosome. Except chromosome 8, the CIPK genes are distributed variably on seven chromosomes.

All the 22 *CaCIPK* genes were mapped onto the seven out of eight chromosomes of chickpea (Fig. 3). Chromosome 1 contains the maximum number of genes viz. *CIPK1, -7, -9, -12, -20*. Chromosomes 2 and 7 harbour four genes each i.e., *CIPK2*, *-*3, *-*8, *-*14 and *CIPK6*, *-17*, *-19*, *-21* respectively. Chromosome 5 contains three genes (*CIPK10*, *-11*, *-13*), that are located very close to each other. The other chromosomes have only two *CIPKs* each. It was observed that the CaCIPK family had undergone gene duplication as nine gene pairs showed segmental duplications, including *CaCIPK1/17*, *CaCIPK2/10, CaCIPK2/18, CaCIPK5/7, CaCIPK10/18, CaCIPK11/14, CaCIPK11/22, CaCIPK14/22,* and *CaCIPK15/18* (Figure S4). In addition to this, four gene pairs (e.g. *CaCIPK12/20, CaCIPK2/14, CaCIPK18/22, CaCIPK10/11*) showed tandem duplications. A ratio of Ka (non-synonymous)/Ks (synonymous substitution) = 1, signifies neutral selection (drift), Ka/Ks < 1 indicates purifying selection, and Ka/Ks > 1 implies positive selection (adaptive evolution)^53^. In our study, the ratio of Ka to Ks for *CaCIPKs* ranged from 0.0068 to 0.1675 (Table S3).

### *Cis*-regulatory elements in *CaCIPK* promoters

**Figure 4:**
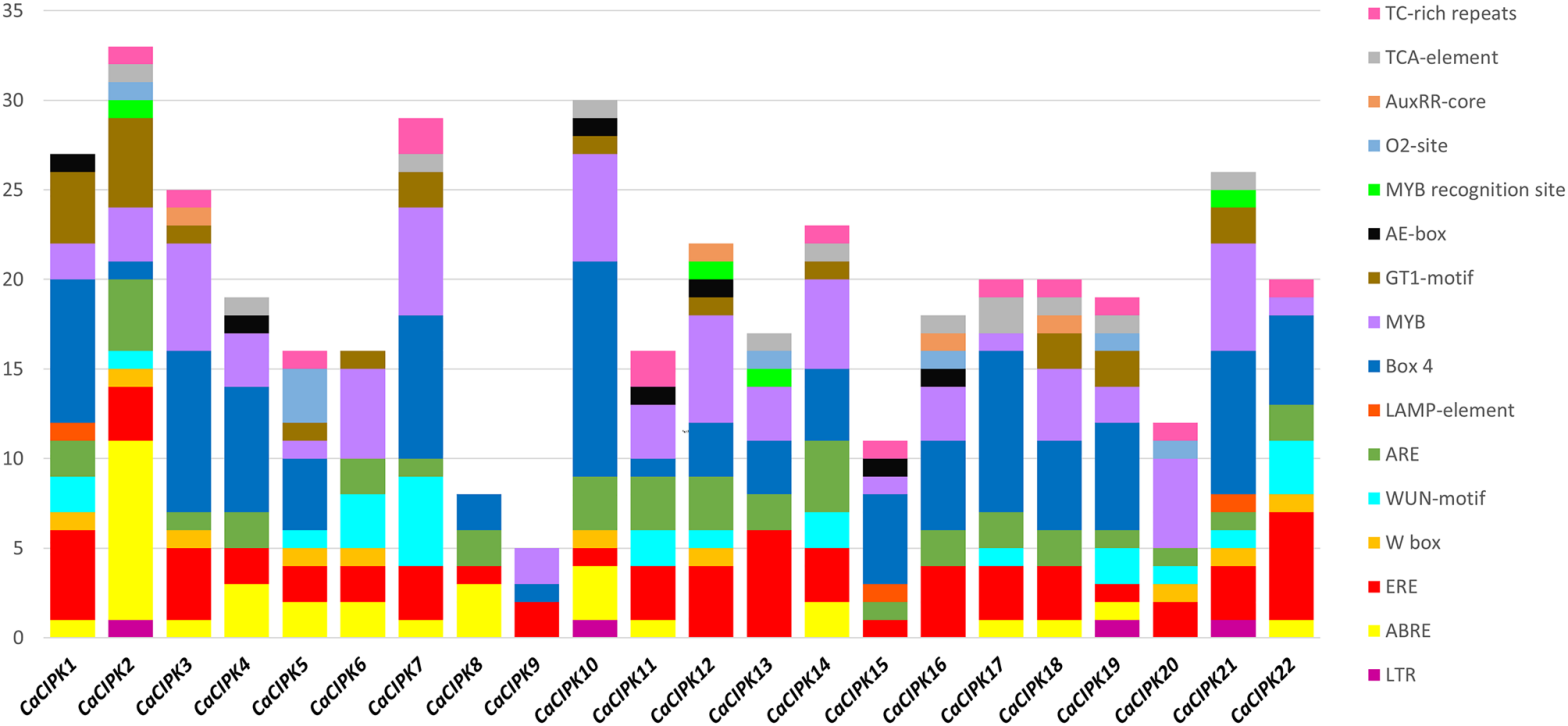
Promoter analysis of chickpea *CIPK* genes. Different *cis*-regulatory elements in the 2 kb upstream region of 22 *CaCIPK* genes are illustrated by different colours in the bar chart. The X-axis represents the name of genes and Y-axis represents the number of *cis*-elements in each promoter. The names of *cis*-regulatory elements are mentioned on the right.

Various *cis*-regulatory elements were found to be unevenly distributed on the promoters of *CIPK* genes in chickpea (Fig. 4). An oxidative stress-responsive element ERE^54^ was present in all *CaCIPKs*. The W box has a role in both biotic and abiotic stress^55^, and was present in *CaCIPK1, -2, -3, -5, -6, -10, -12, -20, -21, -22*. The MYB was present in all the *CaCIPK* genes except *CaCIPK8*. The O_2_ site involved in zein metabolism and circadian motif^54^, was present in only six *CaCIPK* genes viz. *CaCIPK2, -5, -13, -16, -19, -20*. Another well-characterized *cis*-element, ABRE involved in abiotic stress, and ABA responsiveness^56,57^, was found in the promoter of 15 *CaCIPK* genes, including *CaCIPK1, -2, -3, -4, -5, -6, -7, -8, -10, -11, -14, -17, -18, -19, -22*. Besides, LTR-motif was one of the least common elements, and only four *CaCIPK* genes (*CaCIPK2*, *-10*, *-19*, *-21*) contain this motif (Table S4).

### Subcellular localization and structure prediction

**Figure 5:**
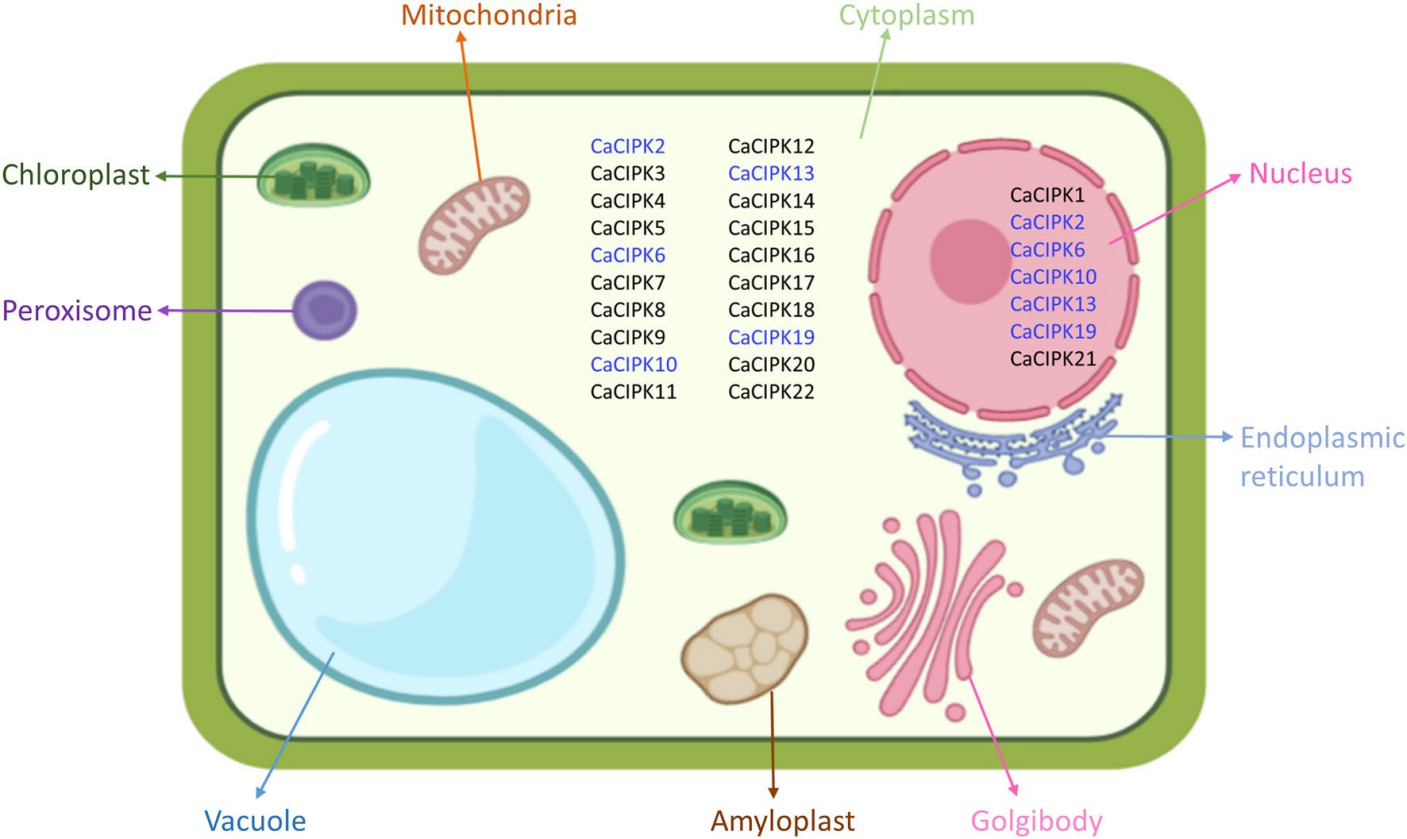
Subcellular localization of chickpea CIPK proteins. Localization was predicted using the CELLO program. Genes located in both cytoplasm and nuclei are indicated by blue font.

In our study, the majority of CaCIPK proteins were found to be localized in the cytoplasm, and only six CaCIPK proteins were localized in the nucleus (Fig. 5). Among 22 CIPKs, five proteins namely CaCIPK2, *-*6, *-*10, *-*13, *-*19 were found to be localized both in the nucleus and cytosol.

**Figure 6:**
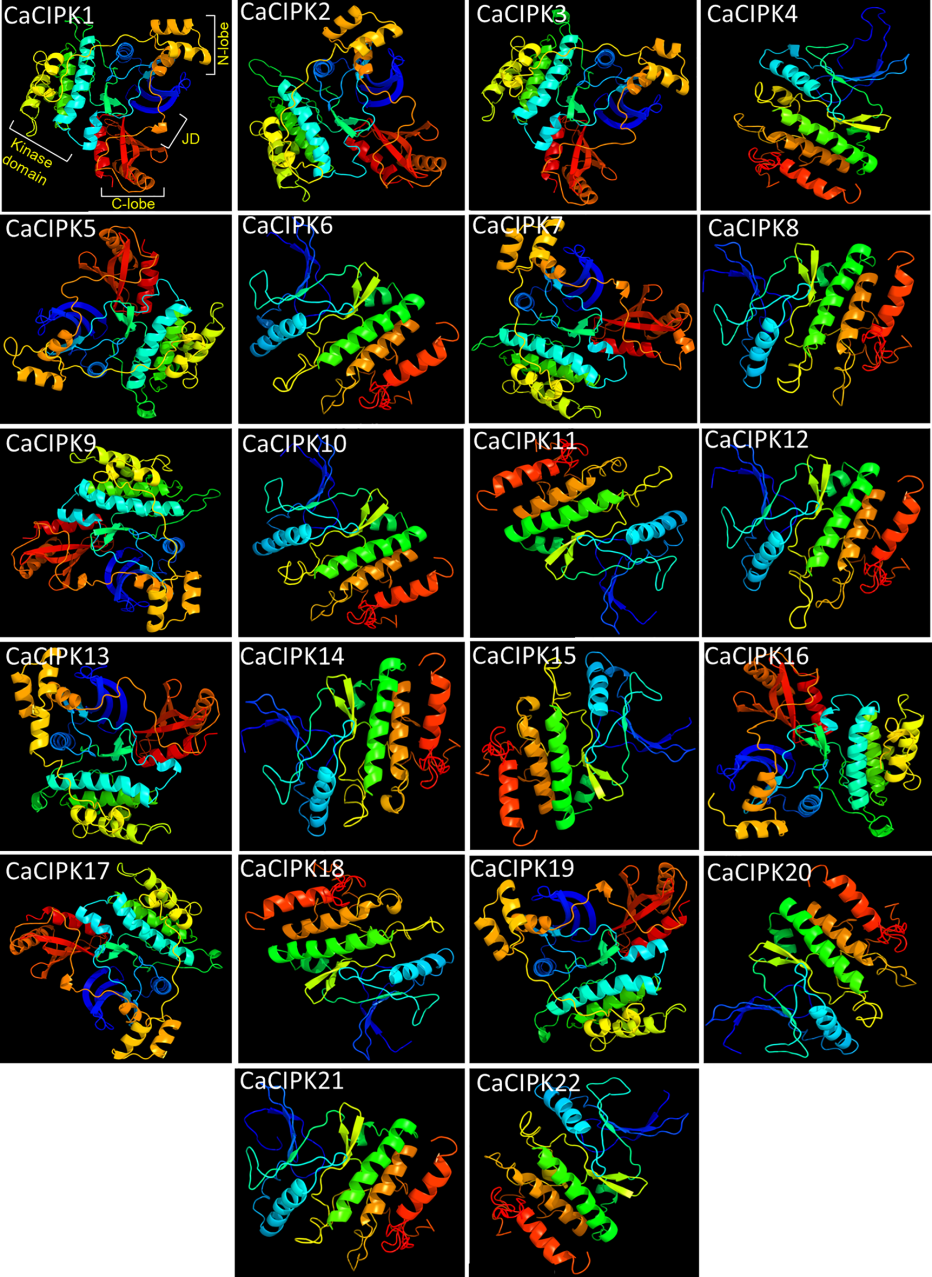
Three-dimensional structure of chickpea CIPK proteins. 3-D structure was generated for all 22 members of chickpea CIPK family using PHYRE2. Each CIPK protein is made up of variable number of α-helix, β-strands, transmembrane helix and disordered region.

The three-dimensional (3D) structures of 22 CaCIPK proteins were modelled with 100% confidence by the single highest scoring template (Fig. 6). A major part of the models was based on the two templates e.g. c6c9dB (Serine/threonine-protein kinase MARK1), and c5ebzF (inhibitor of nuclear factor kappa-b kinase subunit alpha), based on raw alignment score which takes into account the sequence and secondary structure similarity, inserts and deletes. The CaCIPK16 showed the maximum coverage (95%), whereas CaCIPK12 showed the least coverage (50%) in the alignment. The identity of the template model c4czuC (belonged to CIPK23) with other CIPKs varied from 52 to 85% (Table S5). All the CaCIPK proteins were found to have comparable numbers of α-helices and β-sheets, ranging from 16 to 21, and 14–17 respectively.

### Interaction patterns between CBL and CIPK proteins in chickpea

**Figure 7:**
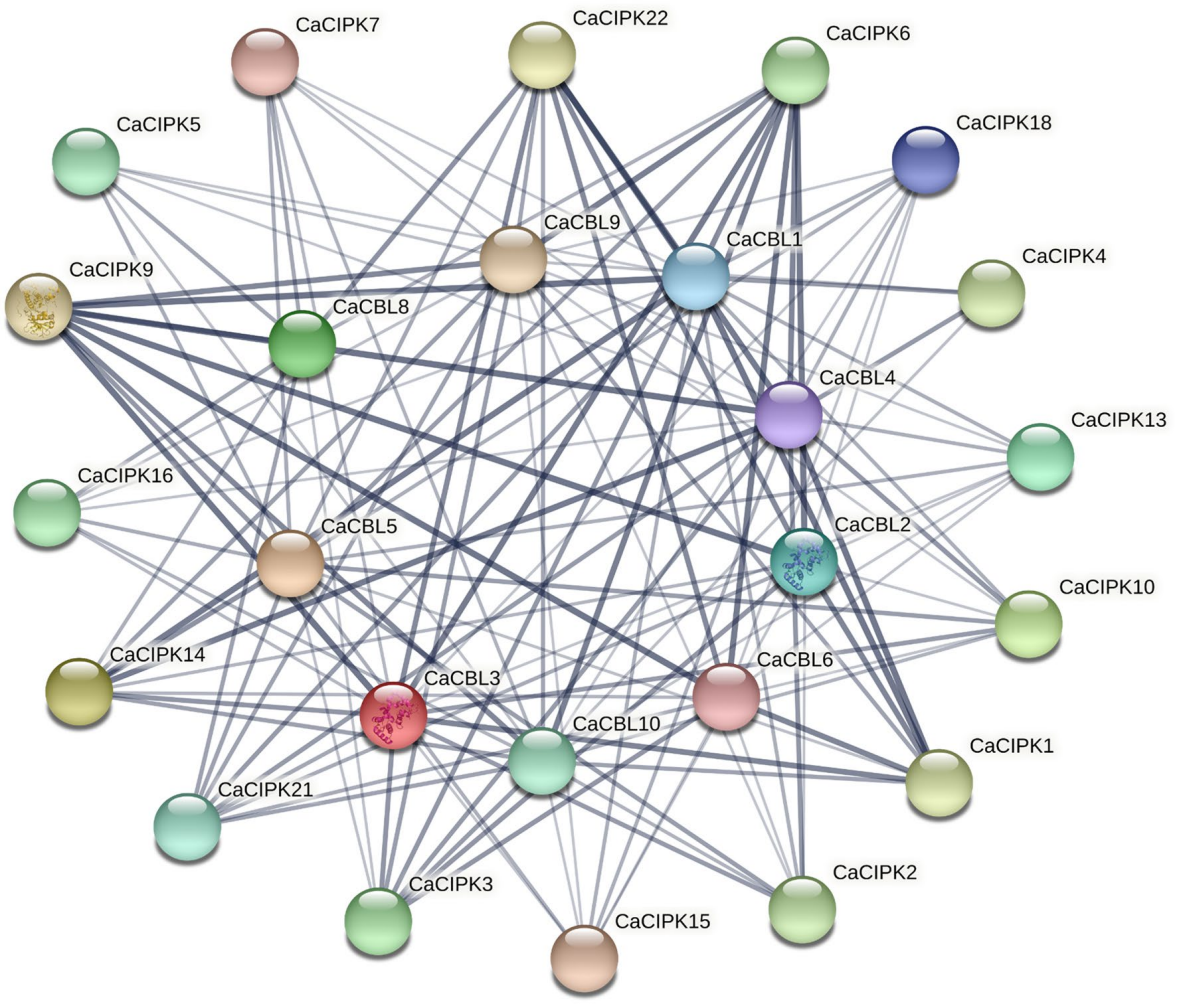
Interaction networks of CaCIPKs and CaCBLs based on chickpea homologs in the STRING database. The best matching sequence of CaCIPK protein with chickpea on the basis of identity, bitscore and e-value has been shown in the figure. Experimental data of CBL and CIPK interaction in chickpea was constructed by using the confidence value > 0.4. The thicker and thinner lines represent strong and weak interaction respectively.

CIPKs are generally activated by interaction with CBLs to perform different functions. Thus, it is crucial to determine the interactions and functional complexes of CBLs and CIPKs in chickpea. Therefore, in silico analysis was performed to analyze the CBL and CIPK interactions in chickpea. A combined score of co-expression, experimentally determined interaction, and automated text mining was used to predict the strength of interaction. A score of less than 0.7 was taken as weak, whereas a score greater than 0.7 was considered as strong interaction. The CaCBL1 showed strong interaction (thicker lines) with CaCIPK1, *-*3, *-*6, *-*9, *-*14 and *-*22, and exhibited weak (thinner lines) interactions with CaCIPK2, *-*4, *-*5, *-*7, *-*10, *-*13, *-*15, *-*18 and *-*21 (Fig. 7). The CaCBL2 showed strong interactions with four CaCIPKs, including CaCIPK3, *-*6, *-*9 and *-*22, and weak interactions with eight CaCIPKs (e.g. CaCIPK1, *-*2, *-*10, *-*13, *-*14, *-*15, *-*18 and *-*21). CaCBL4 showed strong interactions with seven CIPKs, namely CaCIPK1, *-*2, *-*6, *-*9, *-*10, *-*14, *-*22. The CaCBL5 interacted with a total of 14 CaCIPKs among which it showed strong interactions with only CaCIPK9 and *-*14 and weak interactions with CaCIPK1, *-*2, *-*3, *-*5, *-*6, *-*10, *-*13, *-*15, *-*16, *-*18, and *-*22. The CaCBL8 was found to interact with nine CaCIPKs, including CaCIPK3, *-*5, *-*6, *-*7, *-*9, *-*14, *-*16, *-*21 and *-*22. The CaCBL9 showed strong interaction with only two CaCIPKs namely CaCIPK9 and CaCIPK6, out of which CaCIPK9 shows homology with AtCIPK23 according to the phylogenetic tree. The CaCBL10 interacts with sixteen CaCIPKs (e.g. CaCIPK1, *-*2, *-*3, *-*4, *-*5, *-*6, *-*7, *-*9, *-*10, *-*13, 14, *-*15, 16, *-*18, *-*21, *-*22), out of those, it showed strong interactions with only two CaCIPKs (CaCIPK6 and CaCIPK9) (Table S6).

### Expression profile of *CIPK* genes in different developmental stages

**Figure 8:**
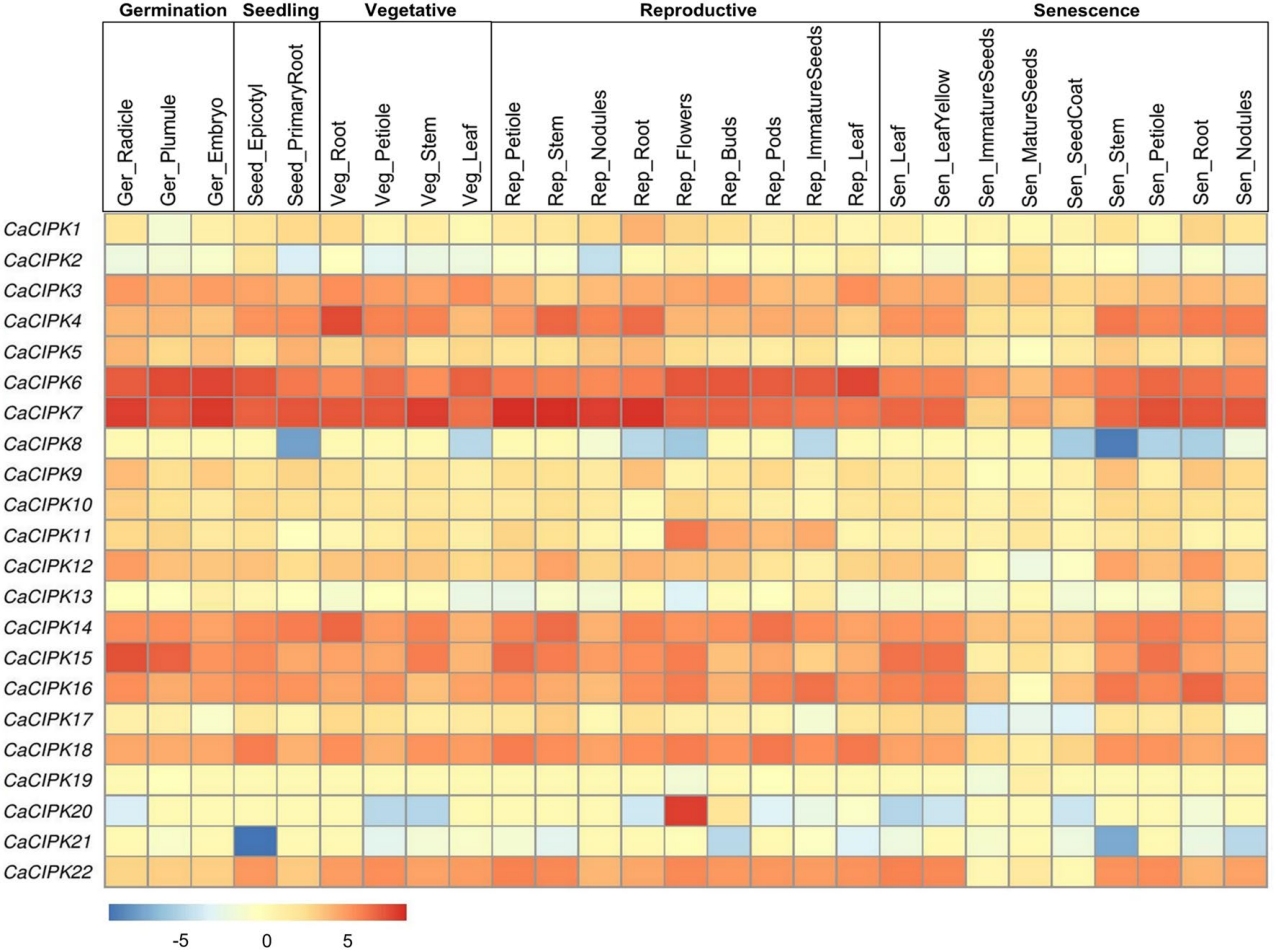
Expression profiles of different *CIPK* genes in different developmental stages of chickpea. The heatmap represents the expression pattern of *CaCIPK* genes in the developmental stages of different tissues such as germination, seedling, vegetative, reproduction and senescence. The genes are named on the left and different tissues/developmental stages are labelled at the top. Scale bar represents the normalised log_2 FPKM values.

The expression analysis of *CaCIPKs* was carried out in 27 tissues of chickpea belonging to different stages i.e. germination stage (radicle, plumule, embryo), seedling stage (epicotyl, primary root), vegetative stage (root, petiole, stem, leaf), reproductive stage (nodules, flowers, buds, pods, immature seeds), and senescence stage (yellow leaf, immature seeds, mature seeds, seed coat, and nodules) (Fig. 8). The *CaCIPK3, -4, -6, -7, -14, -15, -16, -18* and *-22* were found to have ubiquitously high expression in all the tissues. Whereas, *CaCIPK2, -8, -13, -17,* and *-21* showed low expression in almost all the tissues.

### Expression profile in different stages of seed development

**Figure 9:**
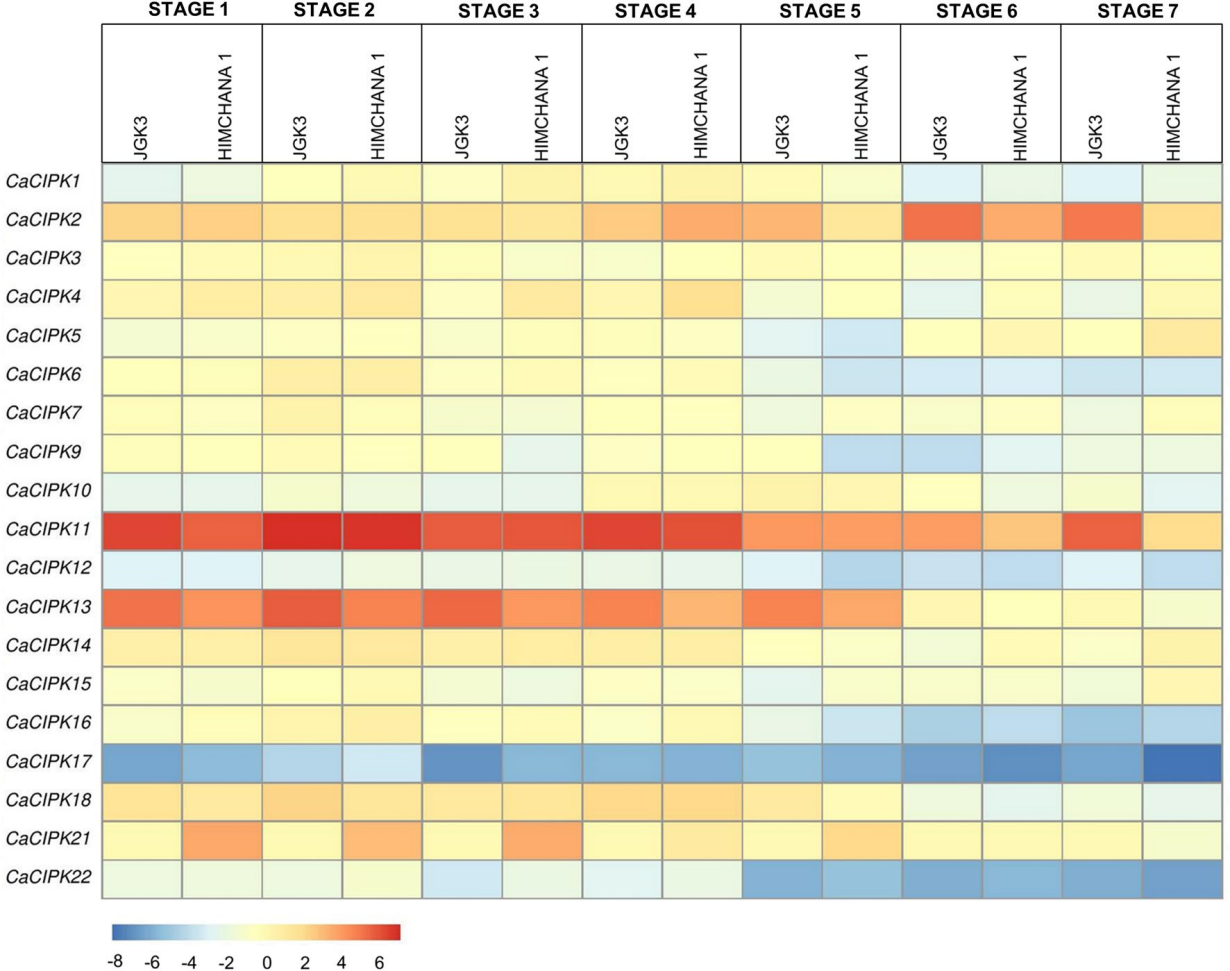
Expression profiles of *CIPK* genes in different stages of seed in chickpea in two cultivars: JGK3 and Himchana1. The heatmap represents the expression pattern for seven stages of seed developmental (S1-S7) in two chickpea cultivars: large-seeded JGK3 and small-seeded Himchana1. The genes are named on the left and stages/cultivars are labelled on top. The scale bar represents the log_2 Fold Change based on FPKM values.

Optimum development of seeds leads to their production in sufficient quantity as well as quality, thereby determining the yield. To understand the role of *CIPKs* in chickpea seed development stages, expression profile was generated with mature leaf as control, and seven different seed stages, representing early-embryogenesis (S1), mid-embryogenesis (S2), late-embryogenesis (S3), mid-maturation (S4-S5), and late-maturation (S6-S7), in two desi cultivars: JGK3 (large-seeded) and Himchana1 (small-seeded) (Fig. 9). Few *CaCIPK* genes, including *CaCIPK2, -11, -13* were ubiquitously expressed during all the seed stages in both the chickpea varieties, however, the level of expression varied (Table S8). *CaCIPK2* expressed highly during S5-S7 in JGK3, and S4 in Himchana1. *CaCIPK11* showed high expression during S1-S5 in both the varieties and during S7 in Himchana1. Similarly, *CaCIPK13* showed significant expression during S1-S5, however, the level of expression was higher in JGK3 than Himchana1. In contrast, *CaCIPK18* and *-21* showed significant expression during S1-S5 in both varieties. Remarkably, *CaCIPK6* and *-16* were upregulated during S1-S4 but downregulated during S5-S7 in both varieties. *CaCIPK10* was upregulated during S4-S5 in both the varieties, but upregulated during S6 only in JGK3. Two *CaCIPK* members, *CaCIPK12* and *-17* were significantly downregulated during all seed stages in both JGK3 and Himchana1.

### qRT-PCR expression analysis under ABA, drought, and salinity stress

**Figure 10:**
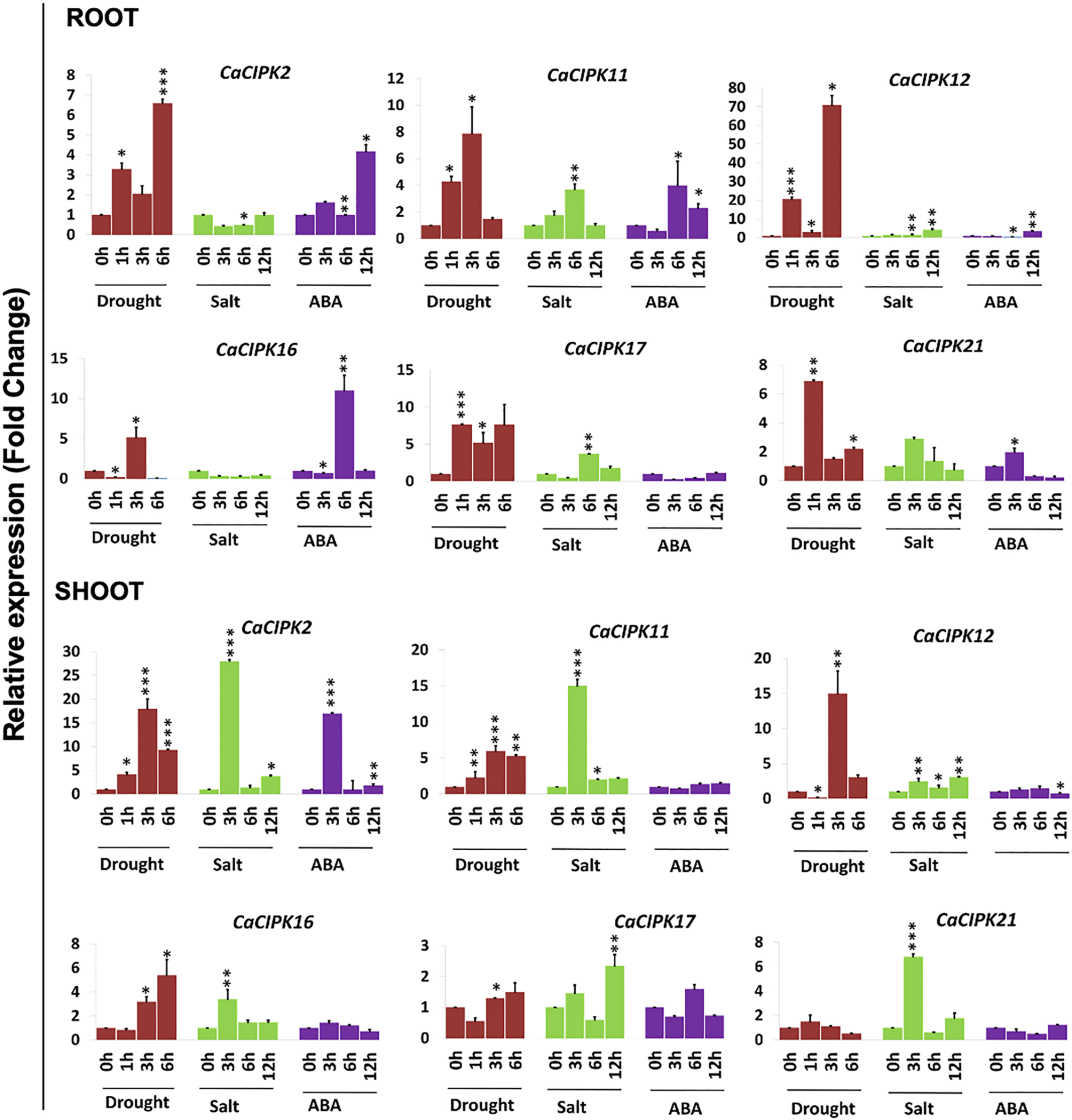
Expression profiles of *CIPK* genes under drought, salt and ABA treatments in chickpea. qRT-PCR analysis was performed to generate the expression profile of *CaCIPK* genes under drought, salt and ABA treatment in root and shoot of desi chickpea (ICC4958). Different treatments and time points are indicated on X-axis and relative expression level of gene is indicated on Y-axis. Each bar represents mean value of three replicates. Standard error among the samples is indicated by error bars. * represents *p*-value < 0.05, ** *p*-value < 0.01 and *** *p*-value < 0.005 for treated samples w.r.t. untreated control.

Abiotic stresses, such as drought and salinity cause the increase in cytosolic Ca^2+^ concentration, that develops a specific “Ca^2+^ signatures”. CIPKs are important components of Ca^2+^ sensing and signaling machinery in plants thus, they are crucial for plant’s adaptation to abiotic stresses. Therefore, to understand the role of CIPKs in abiotic stress signaling in chickpea, qRT-PCR analysis was performed for few selected genes including i.e., *CaCIPK2*, *CaCIPK11*, *CaCIPK12*, *CaCIPK16*, *CaCIPK17* and *CaCIPK21* under the treatment of abiotic stresses such as drought, salt stress and ABA. Expression data was obtained at different time points of treatment in root and shoot tissues, separately. Based on the expression fold change value ≥ 1.5 w.r.t. untreated control, all six *CaCIPK* genes showed differential expression under one or more stress conditions (Fig. 10; Table S9). In roots, all six genes expressed differentially under drought condition. *CaCIPK2, 12, 17* and *21* were significantly up-regulated upon 1 h, 3 h and 6 h of drought treatment, while *CaCIPK11* was up-regulated after 1 h and 3 h of drought treatment. *CaCIPK16* was induced after 3 h of drought treatment, while it was down-regulated after 1 h and 6 h drought treatment. These six *CaCIPK* genes were also found to be differentially expressed under ABA treatment. *CaCIPK2, 11, 12, 16* and *21* were up-regulated at one or more time points. Interestingly, *CaCIPK17* which was ubiquitously expressed under drought stress was found to be down-regulated under ABA treatment (Fig. 10). Furthermore, *CaCIPK* genes showed differential expression in one or more stress conditions in shoot. Like root, all six genes showed differential expression under drought and salt stress conditions in shoot. Interestingly, all these genes were up-regulated and none was down-regulated in both the stress conditions. However, only *CaCIPK21* was down-regulated under ABA treatment (Fig. 10).

## Discussion

A total of 26, and 33 CIPK members have been reported in *Arabidopsis* and rice respectively^58^. In this study, 22 *CaCIPK* genes in chickpea were identified after comprehensive analysis, which is comparable with wheat, tomato, and canola having 20, 22, and 23 *CIPKs* genes respectively^59–61^. Similar isoelectric point amongst CaCIPK proteins indicates that they can function in similar microenvironments. Similar structural features, and functional pI had been reported previously in CIPKs of other plants such as *P. mume*^62^, *C. annum*^63^, and *B. napus*^61^. This suggests that plant CIPKs are highly conserved in terms of sequence, and structure which indicates their identical mode of action. All the 22 *CaCIPK* genes in chickpea were divided into two groups (intron-rich and intron-poor) which has also been reported in different plant species, including *Arabidopsis*^64^, rice^65^, soybean^66^, wild sugarcane^67^, and wheat^59^. These findings suggest that the *CIPK* gene family is structurally conserved across the plant kingdom. The CIPK kinase domain contains an ATP binding site and an activation loop. During CIPK activity, the stabilization of substrates at the active site is regulated by the phosphorylation of the activation loop^68^. The activation loop is located between the conserved ‘DFG’ and ‘APE’ amino acid residues. However, in our analysis, few variations were observed in the conserved short motifs. Further investigations are required to assess the effect of these changes on the functions of the activation loop. In CIPKs, the NAF motif mediates the interaction between CIPK and CBL, and the FISL motif maintains the kinase in an inactive state under normal conditions, thereby acting as an autoinhibitory domain^13,14^. The PPI domain is required for the interaction of CIPKs with protein phosphatase 2C (PP2C)^69^. The absence of motif 6 and motif 10 in CaCIPK4 may explain its presence in a separate phylogenetic clade. A similar motif composition of proteins of the same clade was also reported in tomato and Saccharum^60,67^. Furthermore, the sequential arrangement and the size of motifs in all the CaCIPK proteins is quite similar, which hints towards their structural conservation, as previously reported in *Prunus mume*^62^. Interestingly, the phylogenetic tree analysis placed all intron-rich CaCIPKs in group I, and intron-poor CaCIPKs in group II. This indicates the evolutionary conservation among intron-rich and intron-poor CIPKs in chickpea, and suggests that they might have evolved as two distinct groups in chickpea. A similar phylogenetic pattern has been observed for CIPKs in other plants such as *Zea mays*^70^, and *Prunus mume*^62^. Remarkably, CaCIPKs were found to be close to CIPKs of *Arabidopsis* and soybean but distantly placed from rice CIPKs. This suggests that the CIPKs might have evolved separately in dicots and monocots. The chromosomal localization analysis suggested that all the genes were unevenly distributed on the seven chromosomes. The uneven distribution pattern of CIPKs in chickpea is consistent with previous studies of CIPKs in tomato, pepper, sorghum, rice, apple, and woody plant^60,62,63,65,71,72^. From the gene duplication analysis, we found that nine *CaCIPK* gene pairs underwent segmental duplication and four underwent tandem duplication. The *CIPK* gene family has been found to exhibit significant gene duplication in plant species like *Arabidopsis*, rice, and *Vitis vinifera*^73–75^. This suggests that gene duplication has been the major driving force behind the evolution and expansion of the *CIPK* gene family in chickpea and other plant species. Additionally, Ka/Ks < 1 for all segmentally duplicated gene pairs inferred that all duplicated genes of the *CaCIPK* family had undergone purifying selection on the whole genome duplication (WGD). The analysis of *cis*-elements in the *CaCIPK* promoters showed that they were associated with different stress responses; including ARE (anaerobic induction), MYB (drought stress), LTR (low-temperature responsiveness), WUN-motif (wound-responsiveness), MYB recognition site (drought stress) and response to phytohormones, such as ERE (ethylene-responsive element), ABRE (abscisic acid responsiveness), W-box (WRKY transcription factor binding site), TCA-element (salicylic acid responsiveness), AuxRR-core (auxin-responsive element), O_2_-site (cellular development), TC-rich repeats (defence and stress responsiveness), and Box4, GT1-motif, LAMP-element (light responsiveness)^76–82^. Overall, it indicates their role in abiotic and biotic stress responses, phytohormone signaling, and plant development.

Till now, the subcellular localization of a few CIPKs in *Arabidopsis* has been reported and they have been localized in the cytoplasm and nucleus^83,84^. However, subcellular localization of the majority of CIPKs in other plants such as rice, maize, and soybean has not been determined. In general, CBLs recruit CIPKs to plasma membranes or tonoplasts to perform the different functions. For example, AtCBL10 recruits AtCIPK24 at the tonoplast to regulate the vacuolar homeostasis of Na^+^^85^. AtCBL1 and AtCBL9 recruit AtCIPK1 to the plasma membrane, whereas, AtCBL2 recruits it to the tonoplast^86,87^. Interestingly, AtCBL1 and AtCBL9 also recruit AtCIPK23 to the plasma membrane^88^. Therefore, future investigation of CaCBL-CaCIPK interactions under different conditions will reveal their exact subcellular localization. Moreover, the 3D structure of CIPKs has not been fully explored in other plants, however, in *Prunus mume*, 3D structures of PmCIPK proteins were predicted, which shared the highest identity with the hypothetical protein c6c9dB^62^, similar to CaCIPK proteins in our study.

The interaction pattern of CaCIPK and CaCBL suggests that each CaCIPK may interact with multiple CaCBLs and vice versa. Such interactions may determine the functional specificity or overlap of chickpea CBL-CIPK complexes, and the condition-specific subcellular localization of CaCIPKs in manner. Similar interaction patterns of CBL-CIPK complexes have been found in different plant species, including *Arabidopsis*^12^ and rice^74^. For instance, Yadav et al. in 2018 discussed about the interaction specificity of CIPK9 with CBL2 and CBL3 in *Arabidopsis*^89^, whereas another study found the involvement of CIPK21 in salt stress through interaction with CBL2 and CBL3^19^. Previous studies also suggest the CBL4-CIPK24 or CBL10-CIPK24 complex formation at plasma or vacuolar membrane separately through which CIPK24 serves as the common downstream target of CBL4 and CBL10 in *Arabidopsis*^85,90,91^. Similarly, potassium transport processes in roots and stomatal guard cells is regulated by CBL1- and CBL9-CIPK23 complexes^92^. This suggests that CBLs and CIPKs make diverse complexes to display functional specificity and synergism across plant species and regulate a diverse array of processes in plants.

In this study, RNA-seq expression analysis of *CaCIPK*s indicate that most *CaCIPK* genes are induced in multiple tissues and developmental stages and might be involved in the regulation of a wide array of processes during different stages of plant development in chickpea. (Table S7). A similar expression pattern has been observed for *CIPKs* in plants such as *Arabidopsis*, rice, and wheat^59,74^. The *AtCIPK19* was highly expressed in pollen grains and pollen tubes and analysis of *atcipk19* mutant and overexpression plants revealed that *AtCIPK19* is required for pollen tube growth and polarity^24^. *AtCIPK6* and its chickpea ortholog have been shown to regulate root development via modulating auxin transport in *Arabidopsis*^26^. Tomato *SlCIPK2* is specifically expressed in floral organs, and through interaction with different SlCBLs and stress-responsive transcription factors regulates stress tolerance^93^. Also, *CIPKs* in *Manihot esculenta*; *MeCIPK16,* and *MeCIPK20* were predominantly expressed in flowers^94^. These findings suggest that *CIPKs* are key regulators of plant development. Also, the role of CIPKs in seed developmental stages has been proposed in plant species like rice and *Phaseolus vulgaris*. Along with *CBLs*, several *CIPK* genes of rice were differentially expressed during the five stages of seed development^74^. In *Phaseolus vulgaris*, *PvCIPK1*, *-2, -3,* and *-5* were expressed only in small and mid-size developing seeds but showed no expression in large developing seeds^95^. Our findings suggest the crucial role of CIPKs in seed development stages of chickpea. Some CIPKs might be involved in regulating all the seed development stages in both varieties, whereas some members might regulate specific seed stages in both varieties or any specific variety. Thus, CIPKs could play an important role in determining chickpea yield. The qRT-PCR analysis under abiotic stress treatment such as drought, salt stress and ABA indicates that while most of the *CaCIPK* genes express constitutively under drought stress while some genes express in in a time specific manner. ABA is known to regulate many facets of plant growth, such as root growth and seed germination^96^. An overlapping expression of genes like *CaCIPK2, 11, 12* and *16* in ABA and drought conditions hint towards their role in root growth to improve water uptake under drought stress via ABA-dependent pathway. ABA pathway is well known to regulate drought and salinity stress response^97^. Several studies have revealed the co-regulation of genes under drought and salinity stress^9,47,49,98^. Consistently, in our study, genes such as *CaCIPK11*, *12*, *17* and *21* which were up-regulated under drought, were also induced under salt stress. Surprisingly, *CaCIPK2* which was ubiquitously expressed under drought stress was down-regulated under salt stress. Salt stress is known to have two components; osmotic and ionic stress^99^. Osmotic component is common in both drought and salt stress, whereas ionic component is unique to salt stress. Possibly, genes like *CaCIPK2* might regulate the salt stress through its ionic component only. However, a detailed functional investigation is required to confirm its function. Our analysis showed that like root, all six genes were differentially expressed under drought and salt stress conditions in shoot. Remarkably, all six genes were up-regulated and none was down-regulated in both the stress conditions. These findings suggest that *CaCIPK* genes could be involved in regulating stomata closure and transpiration rate under drought and salinity stresses via ABA signaling pathway. In future, these potential candidate genes could be used in genetic engineering for improving abiotic stress tolerance in chickpea. Previously, several *CIPK* genes which exhibit differential expression pattern under abiotic stresses were characterized functionally and shown to regulate abiotic stress responses in plants. For example, AtCIPK21 was up-regulated by salt, mannitol, ABA, and drought conditions. The *cipk21* knockout mutant plants showed hypersensitivity to osmotic and salt stress. This hypersensitive phenotype could be rescued upon functional complementation of *cipk21* with CIPK21 coding ORF. This confirmed that CIPK21 functions as positive regulator of osmotic and salt stress^19^. Similar functional response was also shown by CIPK1 to osmotic stress and ABA in *Arabidopsis*^86^. Salt overly sensitive (SOS) pathway is well characterized in *Arabidopsis*, where membrane-localized *AtCBL4* (SOS3) interact and activate *AtCIPK24* (SOS2), which in turn regulated membrane-localized Na^+^/H^+^ antiporter (SOS1) to confer salt tolerance^18^. Alternatively, *AtCIPK8*, the homolog of *AtCIPK24* interacts with *AtCBL10* and activates SOS1 to confer salt tolerance^100^. Besides *Arabidopsis*, CIPKs have been implicated in regulation of abiotic stress tolerance in crop plants. For instance, *OsCIPK23* RNAi rice plants exhibit a hypersensitive response to drought stress. Maize *ZmCIPK8* has been shown to regulate the expression of stress-related genes, and it improves drought tolerance in tobacco^101^. In *Hordeum brevisubulatum*, *HbCIPK2* is up-regulated by salt, drought, and ABA treatment, and its overexpression confers salt tolerance in *Arabidopsis sos2-1* mutant and wild-type plants^102^. These findings indicate the CIPKs act as positive regulators of abiotic stress responses both in model plant *Arabidopsis* and crop plants. Strikingly, *CaCIPK2* and *CaCIPK11* were strongly induced during seed developmental stages, and under drought stress and ABA treatment. Careful analysis showed that promoters of these genes also contain one or more ABRE element. Seed development and drought stress are known to be interconnected and ABA simultaneously regulate these responses. Thus, *CaCIPK2* and *CaCIPK11* probably by regulating drought response might control seed development via ABA dependent pathway in chickpea. Such genes could be crucial in improving abiotic stress tolerance and crop yield; therefore, they will be of great biotechnological importance.

Upon comparing the expression pattern of *CaCIPKs* to a closely related legume *Cajanus cajan*^103^, we observed a similar trend like that of chickpea. In *Cajanus cajan*, multiple *CcCIPK* genes were involved in different developmental stages, with exceptions like *CcCIPK19* was specifically expressed in seed in reproductive stage (Figure S5). We further investigated the expression pattern of *CcCIPKs* in response to heat and salt stress, which indicated that some genes are specifically expressed in leaves, root and shoot (Figure S6). For instance, *CcCIPK1*, *-5* and *-8* displayed high expression level in leaves in response to heat stress. Many *CcCIPK* genes like *CcCIPK2*, *-6*, *-7*, *-20*, *-21*, *-26*, *-27* responded higher to prolonged heat stress of 3 h. Even the two varieties of pigeonpea namely ICP1071 (salt susceptible) and ICP7 (salt tolerant) showed distinct expression pattern in response to salt stress, whereas two genes *CcCIPK18* and *CcCIPK19* exhibited the same behaviour in both the variants. Together, these results further verify that *CIPKs* in pigeonpea play multiple roles in development as well as stress responses similar to chickpea.

### Conclusion

CIPKs have been studied at the genome-wide scale in diverse plant species, however, in-depth study of the *CIPK* gene family was missing in important legume crop chickpea. Therefore, in this study, genome-wide identification and characterization of the *CIPK* gene family was carried out in chickpea and a total of 22 *CIPK* genes were unearthed. Gene and protein structure analysis indicated structural conservation among chickpea CIPKs and homology with other plant species. Phylogenetic analysis suggested that chickpea CIPKs have evolved from common dicot ancestors and gene duplication is the major driving force behind their evolution and expansion. Subcellular analysis showed that the CIPK proteins are majorly located in the nucleus and cytoplasm. In-silico interaction analysis revealed various specific and overlapping functional complex formations between CBLs and CIPKs which could be tested functionally in the future. Expression analysis during various developmental stages indicated that *CIPKs* are expressed in a wide range of tissue/organs and could play an important role in their development. Promoter and expression analysis of the *CIPK* gene family strongly suggest their role in abiotic stress signaling and seed development stages in chickpea. Thus, this study provides a useful platform for detailed functional characterization of the CIPK family in chickpea and other legume crops.

## Supplementary Information

Supplementary Information.

## Data Availability

RNA-Seq data used in this study is available at Short Read Archive (SRA) of NCBI with accession number SRP121085, SRP072563 and SRP072564.
