# Supplementary material for: Molecular and expression analysis indicate the role of CBL interacting protein kinases (CIPKs) in abiotic stress signaling and development in chickpea
Source: Sci Rep. 2022 Oct 7;12:16862. doi: 10.1038/s41598-022-20750-2 (PMC9546895; doi:10.1038/s41598-022-20750-2)
Supplement: Supplementary file 1 — Supplementary Information. [file 41598_2022_20750_MOESM1_ESM.docx]

**Table S1:** List of primers used in qRT-PCR analysis of chickpea CIPK genes.

| **Purpose** | **Primer** | **Sequence** |
| --- | --- | --- |
| \| qRT-PCR \| \| --- \| | *CaEF1α* F | 5' TCCACCACTTGGTCGTTTTG 3' |
| qRT-PCR | *CaEF1α* R | 5' CTTAATGACACCGACAGCAACAG 3' |
| qRT-PCR | *CaCIPK2* F | 5' CCGGTTGTGCCTGTGAGTAT 3' |
| qRT-PCR | *CaCIPK2* R | 5' AGCAATCTCTTCCAGCCTCG 3' |
| qRT-PCR | *CaCIPK11* F | 5' TGTCTCCACGAAGTTCTCGC 3' |
| qRT-PCR | *CaCIPK11* R | 5' AACCGACCCTTTGCGATCTT 3' |
| qRT-PCR | *CaCIPK12* F | 5' GATGATTGACGCCGCCTTT 3' |
| qRT-PCR | *CaCIPK12* R | 5' AATCGTGAGGGCCAAGAAGA 3' |
| qRT-PCR | *CaCIPK16* F | 5' TCACACAACCAACATTCCTTCAC 3' |
| qRT-PCR | *CaCIPK16* R | 5' GGAATTTTGGAAGGTTGTTGAGA 3' |
| qRT-PCR | *CaCIPK17* F | 5' CCAGGATAACGATGACGGGG 3' |
| qRT-PCR | *CaCIPK17* R | 5' ATACGGATTGGCGATCCTGG 3' |
| qRT-PCR | *CaCIPK21* F | 5' AGAAAACTTGCTCCTTGACAGC 3' |
| qRT-PCR | *CaCIPK21* R | 5' GGAGCCACATAGCACGGGGAAC 3' |

**Table S2:** Sequence logo of different motifs identified through MEME.

| **Logo** | **E-value** | **Sites** | **Width** |
| --- | --- | --- | --- |
| 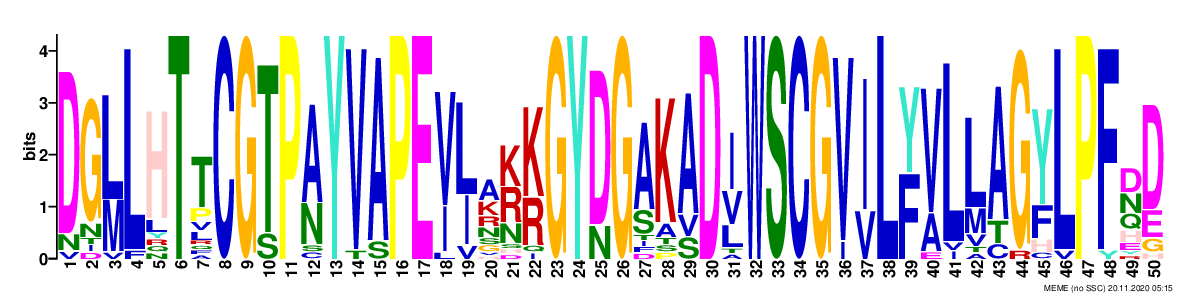 | 1.1e-797 | 22 | 50 |
| 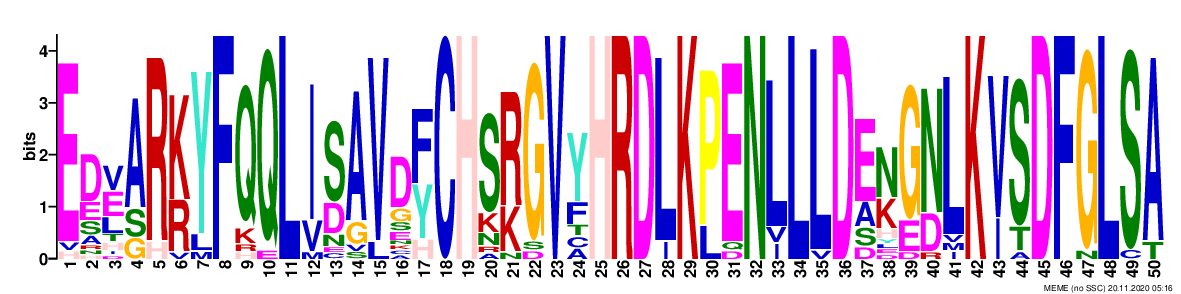 | 5.1e-768 | 22 | 50 |
| 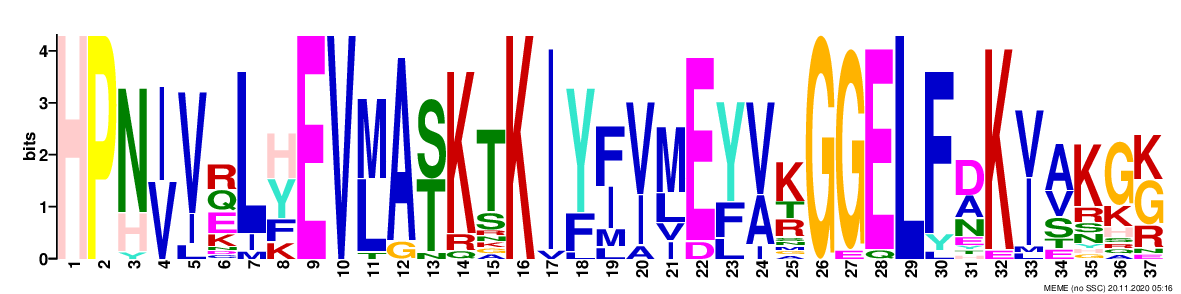 | 2.0e-441 | 22 | 37 |
| 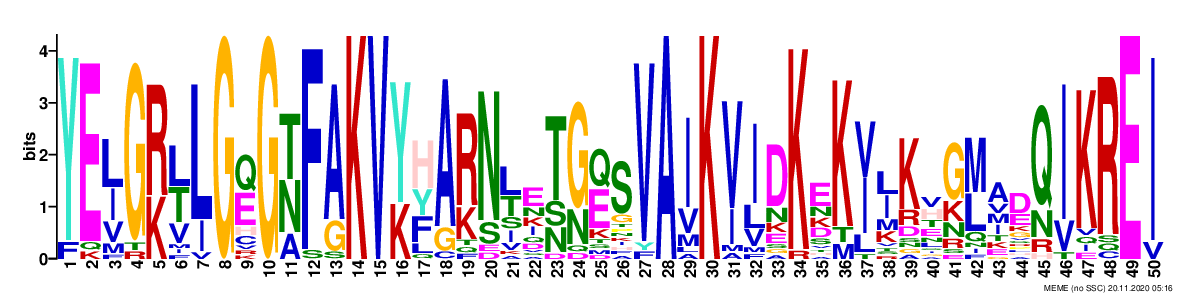 | 3.1e-502 | 22 | 50 |
| 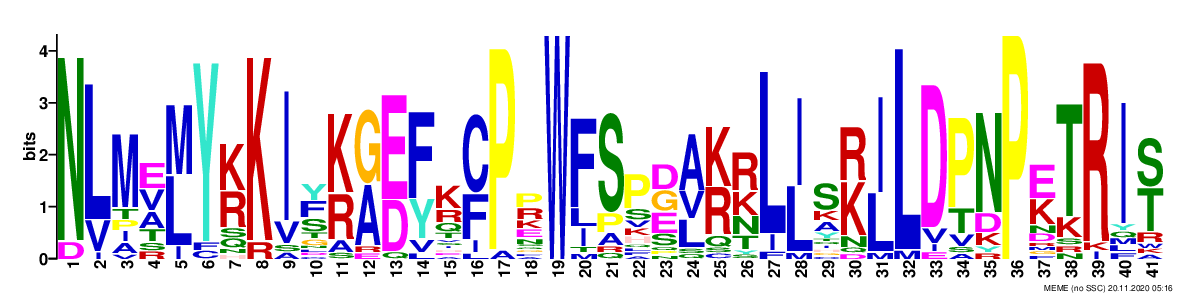 | 5.7e-409 | 22 | 41 |
| 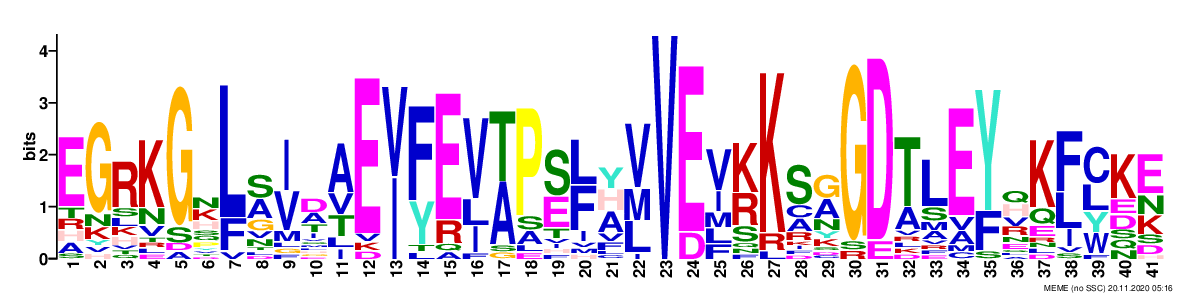 | 2.7e-254 | 21 | 41 |
| 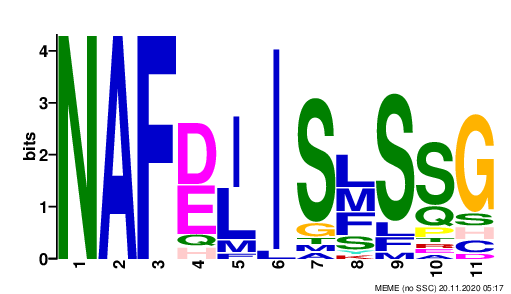 | 4.4e-097 | 22 | 11 |
| 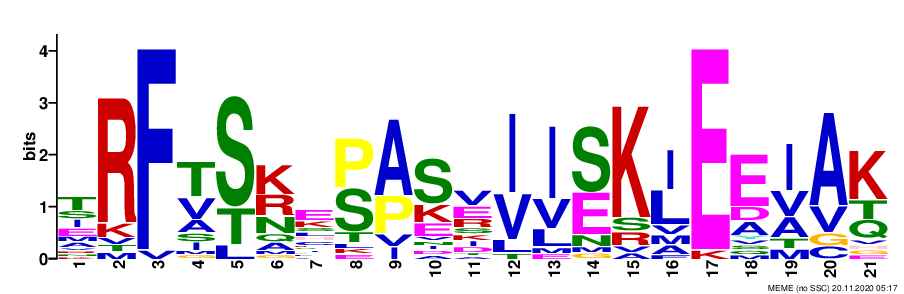 | 2.7e-092 | 22 | 21 |
| 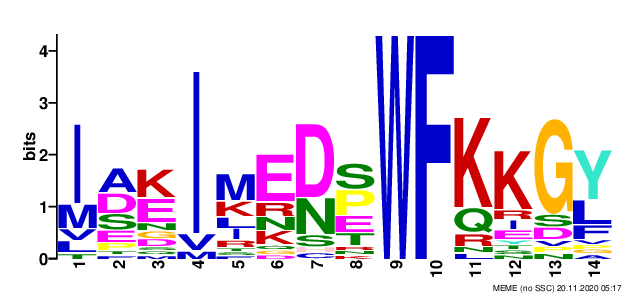 | 7.1e-084 | 22 | 14 |
| 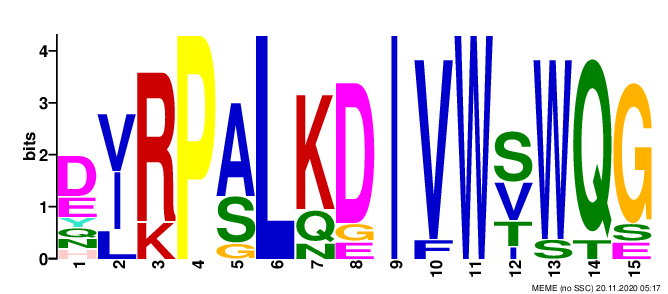 | 3.1e-065 | 10 | 15 |

**Table S3:** List of segmentally duplicated gene pairs.

| **Collinear gene pair** | | | | |
| --- | --- | --- | --- | --- |
| **Gene name** | **Gene name** | **Ka** | **Ks** | **Ka/Ks** |
| *CaCIPK7* | *CaCIPK5* | 0.1373 | 1.7532 | 0.0783 |
| *CaCIPK1* | *CaCIPK17* | 0.1356 | 0.8096 | 0.1675 |
| *CaCIPK2* | *CaCIPK18* | 0.2566 | 5.4846 | 0.0468 |
| *CaCIPK14* | *CaCIPK22* | 0.3502 | 2.2212 | 0.1577 |
| *CaCIPK2* | *CaCIPK10* | 0.1562 | 1.1211 | 0.1394 |
| *CaCIPK14* | *CaCIPK11* | 0.1903 | 2.101 | 0.0906 |
| *CaCIPK18* | *CaCIPK10* | 0.2768 | 2.8293 | 0.0978 |
| *CaCIPK22* | *CaCIPK11* | 0.3208 | 47.4983 | 0.0068 |
| *CaCIPK18* | *CaCIPK15* | 0.1194 | 0.9769 | 0.1222 |

**Table S4:** List of cis-regulatory elements identified in the promoter regions of *CaCIPK* genes.

|  | ***CaCIPK1*** | ***CaCIPK2*** | ***CaCIPK3*** | ***CaCIPK4*** | ***CaCIPK5*** | ***CaCIPK6*** | ***CaCIPK7*** | ***CaCIPK8*** | ***CaCIPK9*** | ***CaCIPK10*** | ***CaCIPK11*** | ***CaCIPK12*** | ***CaCIPK13*** | ***CaCIPK14*** | ***CaCIPK15*** | ***CaCIPK16*** | ***CaCIPK17*** | ***CaCIPK18*** | ***CaCIPK19*** | ***CaCIPK20*** | ***CaCIPK21*** | ***CaCIPK22*** |
| --- | --- | --- | --- | --- | --- | --- | --- | --- | --- | --- | --- | --- | --- | --- | --- | --- | --- | --- | --- | --- | --- | --- |
| **LTR** |  | 1 |  |  |  |  |  |  |  | 1 |  |  |  |  |  |  |  |  | 1 |  | 1 |  |
| **ABRE** | 1 | 10 | 1 | 3 | 2 | 2 | 1 | 3 |  | 3 | 1 |  |  | 2 |  |  | 1 | 1 | 1 |  |  | 1 |
| **ERE** | 5 | 3 | 4 | 2 | 2 | 2 | 3 | 1 | 2 | 1 | 3 | 4 | 6 | 3 | 1 | 4 | 3 | 3 | 1 | 2 | 3 | 6 |
| **W box** | 1 | 1 | 1 |  | 1 | 1 |  |  |  | 1 |  | 1 |  |  |  |  |  |  |  | 1 | 1 | 1 |
| **WUN-motif** | 2 | 1 |  |  | 1 | 3 | 5 |  |  |  | 2 | 1 |  | 2 |  |  | 1 |  | 2 | 1 | 1 | 3 |
| **ARE** | 2 | 4 | 1 | 2 |  | 2 | 1 | 2 |  | 3 | 3 | 3 | 2 | 4 | 1 | 2 | 2 | 2 | 1 | 1 | 1 | 2 |
| **LAMP-element** | 1 |  |  |  |  |  |  |  |  |  |  |  |  |  | 1 |  |  |  |  |  | 1 |  |
| **Box 4** | 8 | 1 | 9 | 7 | 4 |  | 8 | 2 | 1 | 12 | 1 | 3 | 3 | 4 | 5 | 5 | 9 | 5 | 6 |  | 8 | 5 |
| **MYB** | 2 | 3 | 6 | 3 | 1 | 5 | 6 |  | 2 | 6 | 3 | 6 | 3 | 5 | 1 | 3 | 1 | 4 | 2 | 5 | 6 | 1 |
| **GT1-motif** | 4 | 5 | 1 |  | 1 | 1 | 2 |  |  | 1 |  | 1 |  | 1 |  |  |  | 2 | 2 |  | 2 |  |
| **AE-box** | 1 |  |  | 1 |  |  |  |  |  | 1 | 1 | 1 |  |  | 1 | 1 |  |  |  |  |  |  |
| **MYB recognition site** |  | 1 |  |  |  |  |  |  |  |  |  | 1 | 1 |  |  |  |  |  |  |  | 1 |  |
| **O2-site** |  | 1 |  |  | 3 |  |  |  |  |  |  |  | 1 |  |  | 1 |  |  | 1 | 1 |  |  |
| **AuxRR-core** |  |  | 1 |  |  |  |  |  |  |  |  | 1 |  |  |  | 1 |  | 1 |  |  |  |  |
| **TCA-element** |  | 1 |  | 1 |  |  | 1 |  |  | 1 |  |  | 1 | 1 |  | 1 | 2 | 1 | 1 |  | 1 |  |
| **TC-rich repeats** |  | 1 | 1 |  | 1 |  | 2 |  |  |  | 2 |  |  | 1 | 1 |  | 1 | 1 | 1 | 1 |  | 1 |

**Table S5:** The confidence, coverage and sequence identities of the homologous relationship of the *CaCIPKs.*

|  |  |  | **Model based on the template** | |  |
| --- | --- | --- | --- | --- | --- |
| **Protein** | **Percentage confidence** | **Percentage coverage** | **Template id** | **Percentage identity with template** | **Percentage identity with c4czuC_** |
| CaCIPK1 | 100 | 92 | c6c9dB_ | 30 | 61 |
| CaCIPK2 | 100 | 91 | c6c9dB_ | 29 | 59 |
| CaCIPK3 | 100 | 93 | c6c9dB_ | 33 | 75 |
| CaCIPK4 | 100 | 59 | c5ebzF_ | 25 | 54 |
| CaCIPK5 | 100 | 93 | c6c9dB_ | 31 | 56 |
| CaCIPK6 | 100 | 57 | c5ebzF_ | 27 | 65 |
| CaCIPK7 | 100 | 92 | c6c9dB_ | 29 | 57 |
| CaCIPK8 | 100 | 54 | c5ebzF_ | 29 | 66 |
| CaCIPK9 | 100 | 90 | c6c9dB_ | 29 | 85 |
| CaCIPK10 | 100 | 54 | c5ebzF_ | 29 | 60 |
| CaCIPK11 | 100 | 57 | c5ebzF_ | 28 | 58 |
| CaCIPK12 | 100 | 50 | c5ebzF_ | 31 | 66 |
| CaCIPK13 | 100 | 91 | c6c9dB_ | 31 | 59 |
| CaCIPK14 | 100 | 58 | c5ebzF_ | 26 | 59 |
| CaCIPK15 | 100 | 54 | c5ebzF_ | 27 | 65 |
| CaCIPK16 | 100 | 95 | c6c9dB_ | 30 | 61 |
| CaCIPK17 | 100 | 93 | c6c9dB_ | 29 | 59 |
| CaCIPK18 | 100 | 54 | c5ebzF_ | 28 | 63 |
| CaCIPK19 | 100 | 93 | c6c9dB_ | 31 | 63 |
| CaCIPK20 | 100 | 56 | c5ebzF_ | 27 | 62 |
| CaCIPK21 | 100 | 57 | c5ebzF_ | 28 | 61 |
| CaCIPK22 | 100 | 58 | c5ebzF_ | 23 | 52 |

**Table S6:** Type and strength of interactions between CBL and CIPK proteins in chickpea

| **Node1** | **Node2** | **Node1_string_id** | **Node2_string_id** | **coexpression** | **experimentally_determined_interaction** | **database_annotated** | **automated_textmining** | **combined_score** |
| --- | --- | --- | --- | --- | --- | --- | --- | --- |
| CaCBL1 | CaCIPK7 | XP_004508968.1 | XP_004486613.1 | 0.044 | 0.2 | 0.047 | 0.282 | 0.406 |
| CaCBL1 | CaCIPK1 | XP_004508968.1 | XP_004488187.1 | 0.044 | 0.847 | 0.047 | 0.602 | 0.937 |
| CaCBL1 | CaCIPK9 | XP_004508968.1 | XP_004488520.1 | 0.044 | 0.843 | 0.047 | 0.696 | 0.95 |
| CaCBL1 | CaCIPK2 | XP_004508968.1 | XP_004490705.1 | 0.044 | 0.395 | 0.047 | 0.289 | 0.555 |
| CaCBL1 | CaCIPK14 | XP_004508968.1 | XP_004490706.1 | 0.044 | 0.777 | 0.047 | 0.576 | 0.902 |
| CaCBL1 | CaCIPK3 | XP_004508968.1 | XP_004491202.1 | 0.044 | 0.282 | 0.047 | 0.623 | 0.72 |
| CaCBL1 | CaCIPK18 | XP_004508968.1 | XP_004492544.1 | 0.044 | 0.395 | 0.047 | 0.289 | 0.555 |
| CaCBL1 | CaCIPK22 | XP_004508968.1 | XP_004492546.1 | 0.044 | 0.777 | 0.047 | 0.564 | 0.899 |
| CaCBL1 | CaCIPK4 | XP_004508968.1 | XP_004498005.1 | 0.044 | 0.445 | 0.047 | 0.474 | 0.698 |
| CaCBL1 | CaCIPK16 | XP_004508968.1 | XP_004498818.1 | 0.044 | 0.2 | 0.047 | 0.282 | 0.406 |
| CaCBL1 | CaCIPK10 | XP_004508968.1 | XP_004500345.1 | 0.044 | 0.395 | 0.047 | 0.289 | 0.555 |
| CaCBL1 | CaCIPK13 | XP_004508968.1 | XP_004500544.1 | 0.044 | 0.395 | 0.047 | 0.289 | 0.555 |
| CaCBL1 | CaCIPK15 | XP_004508968.1 | XP_004506437.1 | 0.044 | 0.395 | 0.047 | 0.289 | 0.555 |
| CaCBL1 | CaCIPK5 | XP_004508968.1 | XP_004507079.1 | 0.044 | 0.2 | 0.047 | 0.282 | 0.406 |
| CaCBL1 | CaCIPK6 | XP_004508968.1 | XP_004507724.1 | 0.044 | 0.485 | 0.047 | 0.681 | 0.83 |
| CaCBL1 | CaCIPK21 | XP_004508968.1 | XP_004507818.1 | 0.044 | 0.355 | 0.047 | 0.543 | 0.695 |
| CaCBL2 | CaCIPK1 | XP_004499340.1 | XP_004488187.1 | 0.044 | 0.678 | 0.047 | 0.521 | 0.84 |
| CaCBL2 | CaCIPK9 | XP_004499340.1 | XP_004488520.1 | 0.044 | 0.838 | 0.047 | 0.696 | 0.949 |
| CaCBL2 | CaCIPK2 | XP_004499340.1 | XP_004490705.1 | 0.044 | 0.197 | 0.047 | 0.29 | 0.411 |
| CaCBL2 | CaCIPK14 | XP_004499340.1 | XP_004490706.1 | 0.044 | 0.291 | 0.047 | 0.326 | 0.506 |
| CaCBL2 | CaCIPK3 | XP_004499340.1 | XP_004491202.1 | 0.044 | 0.449 | 0.047 | 0.519 | 0.726 |
| CaCBL2 | CaCIPK18 | XP_004499340.1 | XP_004492544.1 | 0.044 | 0.197 | 0.047 | 0.29 | 0.411 |
| CaCBL2 | CaCIPK22 | XP_004499340.1 | XP_004492546.1 | 0.044 | 0.64 | 0.047 | 0.478 | 0.805 |
| CaCBL2 | CaCIPK13 | XP_004499340.1 | XP_004500544.1 | 0.044 | 0.197 | 0.047 | 0.29 | 0.411 |
| CaCBL2 | CaCIPK15 | XP_004499340.1 | XP_004506437.1 | 0.044 | 0.197 | 0.047 | 0.29 | 0.411 |
| CaCBL2 | CaCIPK10 | XP_004499340.1 | XP_004500345.1 | 0.044 | 0.197 | 0.047 | 0.29 | 0.411 |
| CaCBL2 | CaCIPK21 | XP_004499340.1 | XP_004507818.1 | 0.044 | 0.199 | 0.047 | 0.488 | 0.576 |
| CaCBL2 | CaCIPK6 | XP_004499340.1 | XP_004507724.1 | 0.044 | 0.756 | 0.047 | 0.621 | 0.904 |
| CaCBL3 | CaCIPK7 | XP_004500149.1 | XP_004486613.1 | 0.044 | 0.168 | 0.047 | 0.404 | 0.487 |
| CaCBL3 | CaCIPK1 | XP_004500149.1 | XP_004488187.1 | 0.044 | 0.678 | 0.047 | 0.521 | 0.84 |
| CaCBL3 | CaCIPK9 | XP_004500149.1 | XP_004488520.1 | 0.044 | 0.838 | 0.047 | 0.696 | 0.949 |
| CaCBL3 | CaCIPK2 | XP_004500149.1 | XP_004490705.1 | 0.044 | 0.197 | 0.047 | 0.623 | 0.687 |
| CaCBL3 | CaCIPK14 | XP_004500149.1 | XP_004490706.1 | 0.044 | 0.291 | 0.047 | 0.62 | 0.721 |
| CaCBL3 | CaCIPK3 | XP_004500149.1 | XP_004491202.1 | 0.044 | 0.449 | 0.047 | 0.521 | 0.727 |
| CaCBL3 | CaCIPK18 | XP_004500149.1 | XP_004492544.1 | 0.044 | 0.197 | 0.047 | 0.29 | 0.411 |
| CaCBL3 | CaCIPK22 | XP_004500149.1 | XP_004492546.1 | 0.044 | 0.64 | 0.047 | 0.478 | 0.805 |
| CaCBL3 | CaCIPK16 | XP_004500149.1 | XP_004498818.1 | 0.044 | 0.168 | 0.047 | 0.404 | 0.487 |
| CaCBL3 | CaCIPK15 | XP_004500149.1 | XP_004506437.1 | 0.044 | 0.197 | 0.047 | 0.29 | 0.411 |
| CaCBL3 | CaCIPK13 | XP_004500149.1 | XP_004500544.1 | 0.044 | 0.197 | 0.047 | 0.29 | 0.411 |
| CaCBL3 | CaCIPK21 | XP_004500149.1 | XP_004507818.1 | 0.044 | 0.199 | 0.047 | 0.521 | 0.603 |
| CaCBL3 | CaCIPK10 | XP_004500149.1 | XP_004500345.1 | 0.044 | 0.197 | 0.047 | 0.623 | 0.687 |
| CaCBL3 | CaCIPK6 | XP_004500149.1 | XP_004507724.1 | 0.044 | 0.756 | 0.047 | 0.621 | 0.904 |
| CaCBL4 | CaCIPK7 | XP_004502645.1 | XP_004486613.1 | 0.044 | 0.2 | 0.047 | 0.294 | 0.416 |
| CaCBL4 | CaCIPK1 | XP_004502645.1 | XP_004488187.1 | 0.044 | 0.847 | 0.047 | 0.602 | 0.937 |
| CaCBL4 | CaCIPK9 | XP_004502645.1 | XP_004488520.1 | 0.044 | 0.843 | 0.047 | 0.696 | 0.95 |
| CaCBL4 | CaCIPK2 | XP_004502645.1 | XP_004490705.1 | 0.044 | 0.395 | 0.047 | 0.546 | 0.716 |
| CaCBL4 | CaCIPK14 | XP_004502645.1 | XP_004490706.1 | 0.044 | 0.777 | 0.047 | 0.559 | 0.898 |
| CaCBL4 | CaCIPK3 | XP_004502645.1 | XP_004491202.1 | 0.044 | 0.282 | 0.047 | 0.587 | 0.693 |
| CaCBL4 | CaCIPK18 | XP_004502645.1 | XP_004492544.1 | 0.044 | 0.395 | 0.047 | 0.289 | 0.555 |
| CaCBL4 | CaCIPK22 | XP_004502645.1 | XP_004492546.1 | 0.044 | 0.777 | 0.047 | 0.564 | 0.899 |
| CaCBL4 | CaCIPK4 | XP_004502645.1 | XP_004498005.1 | 0.044 | 0.445 | 0.047 | 0.474 | 0.698 |
| CaCBL4 | CaCIPK16 | XP_004502645.1 | XP_004498818.1 | 0.044 | 0.2 | 0.047 | 0.294 | 0.416 |
| CaCBL4 | CaCIPK10 | XP_004502645.1 | XP_004500345.1 | 0.044 | 0.395 | 0.047 | 0.546 | 0.716 |
| CaCBL4 | CaCIPK13 | XP_004502645.1 | XP_004500544.1 | 0.044 | 0.395 | 0.047 | 0.289 | 0.555 |
| CaCBL4 | CaCIPK5 | XP_004502645.1 | XP_004507079.1 | 0.044 | 0.2 | 0.047 | 0.282 | 0.406 |
| CaCBL4 | CaCIPK15 | XP_004502645.1 | XP_004506437.1 | 0.044 | 0.395 | 0.047 | 0.289 | 0.555 |
| CaCBL4 | CaCIPK21 | XP_004502645.1 | XP_004507818.1 | 0.044 | 0.355 | 0.047 | 0.543 | 0.695 |
| CaCBL4 | CaCIPK6 | XP_004502645.1 | XP_004507724.1 | 0.044 | 0.485 | 0.047 | 0.681 | 0.83 |
| CaCBL5 | CaCIPK7 | XP_004503610.1 | XP_004486613.1 | 0.044 | 0.168 | 0.047 | 0.503 | 0.572 |
| CaCBL5 | CaCIPK1 | XP_004503610.1 | XP_004488187.1 | 0.044 | 0.198 | 0.047 | 0.327 | 0.442 |
| CaCBL5 | CaCIPK9 | XP_004503610.1 | XP_004488520.1 | 0.044 | 0.201 | 0.047 | 0.644 | 0.706 |
| CaCBL5 | CaCIPK2 | XP_004503610.1 | XP_004490705.1 | 0.044 | 0.168 | 0.047 | 0.62 | 0.673 |
| CaCBL5 | CaCIPK14 | XP_004503610.1 | XP_004490706.1 | 0.044 | 0.198 | 0.047 | 0.698 | 0.749 |
| CaCBL5 | CaCIPK3 | XP_004503610.1 | XP_004491202.1 | 0.044 | 0.168 | 0.047 | 0.335 | 0.428 |
| CaCBL5 | CaCIPK18 | XP_004503610.1 | XP_004492544.1 | 0.044 | 0.168 | 0.047 | 0.478 | 0.551 |
| CaCBL5 | CaCIPK22 | XP_004503610.1 | XP_004492546.1 | 0.044 | 0.198 | 0.047 | 0.519 | 0.601 |
| CaCBL5 | CaCIPK16 | XP_004503610.1 | XP_004498818.1 | 0.044 | 0.168 | 0.047 | 0.503 | 0.572 |
| CaCBL5 | CaCIPK10 | XP_004503610.1 | XP_004500345.1 | 0.044 | 0.168 | 0.047 | 0.62 | 0.673 |
| CaCBL5 | CaCIPK13 | XP_004503610.1 | XP_004500544.1 | 0.044 | 0.168 | 0.047 | 0.478 | 0.551 |
| CaCBL5 | CaCIPK15 | XP_004503610.1 | XP_004506437.1 | 0.044 | 0.168 | 0.047 | 0.478 | 0.551 |
| CaCBL5 | CaCIPK5 | XP_004503610.1 | XP_004507079.1 | 0.044 | 0.168 | 0.047 | 0.503 | 0.572 |
| CaCBL5 | CaCIPK21 | XP_004503610.1 | XP_004507818.1 | 0.044 | 0.168 | 0.047 | 0.581 | 0.639 |
| CaCBL5 | CaCIPK6 | XP_004503610.1 | XP_004507724.1 | 0.044 | 0.168 | 0.047 | 0.622 | 0.675 |
| CaCBL6 | CaCIPK10 | XP_004486376.1 | XP_004500345.1 | 0.044 | 0.197 | 0.047 | 0.29 | 0.411 |
| CaCBL6 | CaCIPK18 | XP_004486376.1 | XP_004492544.1 | 0.044 | 0.197 | 0.047 | 0.29 | 0.411 |
| CaCBL6 | CaCIPK15 | XP_004486376.1 | XP_004506437.1 | 0.044 | 0.197 | 0.047 | 0.29 | 0.411 |
| CaCBL6 | CaCIPK2 | XP_004486376.1 | XP_004490705.1 | 0.044 | 0.197 | 0.047 | 0.29 | 0.411 |
| CaCBL6 | CaCIPK13 | XP_004486376.1 | XP_004500544.1 | 0.044 | 0.197 | 0.047 | 0.29 | 0.411 |
| CaCBL6 | CaCIPK14 | XP_004486376.1 | XP_004490706.1 | 0.044 | 0.291 | 0.047 | 0.326 | 0.506 |
| CaCBL6 | CaCIPK21 | XP_004486376.1 | XP_004507818.1 | 0.044 | 0.199 | 0.047 | 0.488 | 0.576 |
| CaCBL6 | CaCIPK3 | XP_004486376.1 | XP_004491202.1 | 0.044 | 0.449 | 0.047 | 0.519 | 0.726 |
| CaCBL6 | CaCIPK22 | XP_004486376.1 | XP_004492546.1 | 0.044 | 0.64 | 0.047 | 0.478 | 0.805 |
| CaCBL6 | CaCIPK1 | XP_004486376.1 | XP_004488187.1 | 0.044 | 0.678 | 0.047 | 0.521 | 0.84 |
| CaCBL6 | CaCIPK6 | XP_004486376.1 | XP_004507724.1 | 0.044 | 0.756 | 0.047 | 0.621 | 0.904 |
| CaCBL6 | CaCIPK9 | XP_004486376.1 | XP_004488520.1 | 0.044 | 0.838 | 0.047 | 0.696 | 0.949 |
| CaCBL8 | CaCIPK7 | XP_004503608.1 | XP_004486613.1 | 0.044 | 0.168 | 0.047 | 0.541 | 0.605 |
| CaCBL8 | CaCIPK9 | XP_004503608.1 | XP_004488520.1 | 0.044 | 0.201 | 0.047 | 0.645 | 0.706 |
| CaCBL8 | CaCIPK14 | XP_004503608.1 | XP_004490706.1 | 0.044 | 0.168 | 0.047 | 0.482 | 0.554 |
| CaCBL8 | CaCIPK3 | XP_004503608.1 | XP_004491202.1 | 0.044 | 0.168 | 0.047 | 0.327 | 0.421 |
| CaCBL8 | CaCIPK22 | XP_004503608.1 | XP_004492546.1 | 0.044 | 0.197 | 0.047 | 0.7 | 0.751 |
| CaCBL8 | CaCIPK16 | XP_004503608.1 | XP_004498818.1 | 0.044 | 0.168 | 0.047 | 0.541 | 0.605 |
| CaCBL8 | CaCIPK6 | XP_004503608.1 | XP_004507724.1 | 0.044 | 0.168 | 0.047 | 0.48 | 0.553 |
| CaCBL8 | CaCIPK5 | XP_004503608.1 | XP_004507079.1 | 0.044 | 0.168 | 0.047 | 0.541 | 0.605 |
| CaCBL8 | CaCIPK21 | XP_004503608.1 | XP_004507818.1 | 0.044 | 0.168 | 0.047 | 0.581 | 0.639 |
| CaCBL9 | CaCIPK7 | XP_004510366.1 | XP_004486613.1 | 0.044 | 0.168 | 0.047 | 0.351 | 0.442 |
| CaCBL9 | CaCIPK1 | XP_004510366.1 | XP_004488187.1 | 0.044 | 0.168 | 0.047 | 0.578 | 0.637 |
| CaCBL9 | CaCIPK9 | XP_004510366.1 | XP_004488520.1 | 0.044 | 0.168 | 0.047 | 0.797 | 0.825 |
| CaCBL9 | CaCIPK2 | XP_004510366.1 | XP_004490705.1 | 0.044 | 0.168 | 0.047 | 0.313 | 0.409 |
| CaCBL9 | CaCIPK14 | XP_004510366.1 | XP_004490706.1 | 0.044 | 0.198 | 0.047 | 0.591 | 0.661 |
| CaCBL9 | CaCIPK3 | XP_004510366.1 | XP_004491202.1 | 0.044 | 0.168 | 0.047 | 0.62 | 0.673 |
| CaCBL9 | CaCIPK18 | XP_004510366.1 | XP_004492544.1 | 0.044 | 0.168 | 0.047 | 0.313 | 0.409 |
| CaCBL9 | CaCIPK22 | XP_004510366.1 | XP_004492546.1 | 0.044 | 0.173 | 0.047 | 0.552 | 0.617 |
| CaCBL9 | CaCIPK4 | XP_004510366.1 | XP_004498005.1 | 0.044 | 0.168 | 0.047 | 0.35 | 0.441 |
| CaCBL9 | CaCIPK16 | XP_004510366.1 | XP_004498818.1 | 0.044 | 0.168 | 0.047 | 0.351 | 0.442 |
| CaCBL9 | CaCIPK10 | XP_004510366.1 | XP_004500345.1 | 0.044 | 0.168 | 0.047 | 0.313 | 0.409 |
| CaCBL9 | CaCIPK13 | XP_004510366.1 | XP_004500544.1 | 0.044 | 0.168 | 0.047 | 0.313 | 0.409 |
| CaCBL9 | CaCIPK15 | XP_004510366.1 | XP_004506437.1 | 0.044 | 0.168 | 0.047 | 0.313 | 0.409 |
| CaCBL9 | CaCIPK5 | XP_004510366.1 | XP_004507079.1 | 0.044 | 0.168 | 0.047 | 0.351 | 0.442 |
| CaCBL9 | CaCIPK6 | XP_004510366.1 | XP_004507724.1 | 0.044 | 0.168 | 0.047 | 0.862 | 0.881 |
| CaCBL9 | CaCIPK21 | XP_004510366.1 | XP_004507818.1 | 0.044 | 0.168 | 0.047 | 0.576 | 0.635 |
| CaCBL10 | CaCIPK7 | XP_004498906.1 | XP_004486613.1 | 0.044 | 0.168 | 0.047 | 0.446 | 0.523 |
| CaCBL10 | CaCIPK1 | XP_004498906.1 | XP_004488187.1 | 0.044 | 0.168 | 0.047 | 0.578 | 0.637 |
| CaCBL10 | CaCIPK9 | XP_004498906.1 | XP_004488520.1 | 0.044 | 0.168 | 0.047 | 0.797 | 0.825 |
| CaCBL10 | CaCIPK2 | XP_004498906.1 | XP_004490705.1 | 0.044 | 0.168 | 0.047 | 0.446 | 0.523 |
| CaCBL10 | CaCIPK14 | XP_004498906.1 | XP_004490706.1 | 0.044 | 0.198 | 0.047 | 0.573 | 0.646 |
| CaCBL10 | CaCIPK3 | XP_004498906.1 | XP_004491202.1 | 0.044 | 0.168 | 0.047 | 0.621 | 0.674 |
| CaCBL10 | CaCIPK18 | XP_004498906.1 | XP_004492544.1 | 0.044 | 0.168 | 0.047 | 0.313 | 0.409 |
| CaCBL10 | CaCIPK22 | XP_004498906.1 | XP_004492546.1 | 0.044 | 0.173 | 0.047 | 0.552 | 0.617 |
| CaCBL10 | CaCIPK4 | XP_004498906.1 | XP_004498005.1 | 0.044 | 0.168 | 0.047 | 0.35 | 0.441 |
| CaCBL10 | CaCIPK16 | XP_004498906.1 | XP_004498818.1 | 0.044 | 0.168 | 0.047 | 0.446 | 0.523 |
| CaCBL10 | CaCIPK15 | XP_004498906.1 | XP_004506437.1 | 0.044 | 0.168 | 0.047 | 0.313 | 0.409 |
| CaCBL10 | CaCIPK13 | XP_004498906.1 | XP_004500544.1 | 0.044 | 0.168 | 0.047 | 0.313 | 0.409 |
| CaCBL10 | CaCIPK5 | XP_004498906.1 | XP_004507079.1 | 0.044 | 0.168 | 0.047 | 0.351 | 0.442 |
| CaCBL10 | CaCIPK10 | XP_004498906.1 | XP_004500345.1 | 0.044 | 0.168 | 0.047 | 0.446 | 0.523 |
| CaCBL10 | CaCIPK21 | XP_004498906.1 | XP_004507818.1 | 0.044 | 0.168 | 0.047 | 0.576 | 0.635 |
| CaCBL10 | CaCIPK6 | XP_004498906.1 | XP_004507724.1 | 0.044 | 0.168 | 0.047 | 0.862 | 0.881 |

**Table S7:** Log2FPKM values of different tissues belonging to different developmental stages.

|  | **Germination** | | | **Seedling** | | **Vegetative** | | | | **Reproductive** | | | | | | | | | **Senescence** | | | | | | | | | |
| --- | --- | --- | --- | --- | --- | --- | --- | --- | --- | --- | --- | --- | --- | --- | --- | --- | --- | --- | --- | --- | --- | --- | --- | --- | --- | --- | --- | --- |
| **Gene** | **Radicle** | **Plumule** | **Embryo** | **Epicotyl** | **Primary Root** | **Root** | **Petiole** | **Stem** | **Leaf** | **Petiole** | **Stem** | **Nodules** | **Root** | **Flowers** | **Buds** | **Pods** | **Immature Seeds** | **Leaf** | | **Leaf** | **Leaf Yellow** | **Immature Seeds** | **Mature Seeds** | **Seed Coat** | **Stem** | **Petiole** | **Root** | **Nodules** |
| CaCIPK1 | 1.774 | -1.490 | 0.852 | 1.826 | 2.665 | 2.642 | 0.458 | 1.110 | 0.090 | 1.444 | 1.674 | 2.572 | 4.090 | 2.789 | 2.340 | 0.853 | 1.103 | 0.338 | | 1.022 | -0.157 | 0.425 | 0.014 | 0.589 | 2.106 | 0.139 | 2.882 | 1.927 |
| CaCIPK2 | -2.335 | -1.788 | -1.297 | 1.637 | -3.686 | -0.624 | -3.235 | -2.493 | -2.310 | -1.002 | -1.040 | -4.632 | 0.096 | 0.929 | -0.319 | -0.205 | -0.017 | 1.240 | | -0.835 | -1.627 | -0.685 | 2.475 | -0.104 | -0.733 | -2.896 | -1.232 | -2.833 |
| CaCIPK3 | 5.066 | 4.230 | 4.853 | 4.678 | 4.042 | 5.303 | 4.862 | 4.639 | 5.373 | 4.149 | 2.681 | 3.724 | 4.355 | 4.392 | 4.841 | 3.804 | 3.546 | 5.432 | | 4.359 | 4.343 | 2.738 | 3.179 | 2.693 | 3.258 | 3.561 | 3.667 | 3.621 |
| CaCIPK4 | 3.878 | 3.817 | 3.366 | 5.266 | 5.292 | 7.585 | 5.762 | 5.757 | 3.698 | 5.065 | 6.681 | 5.816 | 6.526 | 3.988 | 3.839 | 4.355 | 4.065 | 3.022 | | 5.198 | 5.201 | 2.021 | 2.061 | 2.156 | 6.164 | 5.549 | 5.946 | 5.839 |
| CaCIPK5 | 3.947 | 2.683 | 3.606 | 2.036 | 4.170 | 2.892 | 4.007 | 1.972 | 2.677 | 1.950 | 2.105 | 3.274 | 3.830 | 2.404 | 1.382 | 1.216 | 2.131 | -0.202 | | 2.239 | 2.351 | 0.733 | -0.597 | 1.271 | 3.206 | 1.870 | 1.806 | 3.732 |
| CaCIPK6 | 6.992 | 7.575 | 7.778 | 7.239 | 6.096 | 5.563 | 6.453 | 5.318 | 6.826 | 5.864 | 5.730 | 5.475 | 5.982 | 7.195 | 7.204 | 7.056 | 7.044 | 7.828 | | 5.808 | 5.787 | 4.633 | 3.447 | 4.923 | 6.176 | 6.625 | 6.308 | 5.919 |
| CaCIPK7 | 7.897 | 7.191 | 8.194 | 6.904 | 7.135 | 7.136 | 7.270 | 7.992 | 6.297 | 8.493 | 8.565 | 7.882 | 8.223 | 6.821 | 6.805 | 6.428 | 6.081 | 6.044 | | 6.608 | 6.630 | 2.815 | 4.524 | 3.266 | 6.721 | 7.380 | 7.280 | 7.242 |
| CaCIPK8 | 0.000 | 0.000 | 0.000 | 0.000 | -7.760 | 0.000 | 0.000 | 0.000 | -5.032 | 0.000 | 0.000 | -1.516 | -5.053 | -5.963 | 0.000 | 0.000 | -4.978 | 0.000 | | 0.000 | 0.000 | 0.000 | 0.000 | -5.855 | -9.172 | -5.371 | -5.668 | -2.107 |
| CaCIPK9 | 3.695 | 2.336 | 3.123 | 2.115 | 2.890 | 2.337 | 0.972 | 1.869 | 1.050 | 2.189 | 2.120 | 1.399 | 3.522 | 0.618 | 1.949 | 2.579 | 0.754 | 2.418 | | 1.675 | 1.844 | -0.383 | 0.049 | 0.895 | 3.618 | 1.219 | 3.336 | 2.669 |
| CaCIPK10 | 3.074 | 2.092 | 1.367 | 2.590 | 2.212 | 1.805 | 1.641 | 1.644 | 1.347 | 1.578 | 2.287 | 1.485 | 0.000 | 2.724 | 1.925 | 0.881 | 0.243 | 1.784 | | 2.210 | 1.808 | 0.690 | 1.479 | 0.422 | 2.664 | 2.421 | 2.011 | 1.838 |
| CaCIPK11 | 2.616 | 2.863 | 1.614 | 1.846 | -0.604 | 0.212 | 1.076 | 2.492 | 0.952 | 2.829 | 2.002 | 0.388 | -0.280 | 6.064 | 4.213 | 3.750 | 4.218 | 0.489 | | 0.985 | 0.923 | 0.786 | 1.800 | 0.366 | 1.471 | 2.277 | 0.451 | 0.353 |
| CaCIPK12 | 4.745 | 3.602 | 3.287 | 3.506 | 2.476 | 3.304 | 3.456 | 3.393 | 2.619 | 3.215 | 4.568 | 2.867 | 3.969 | 3.556 | 3.287 | 1.981 | 0.794 | 2.891 | | 3.381 | 3.281 | -0.179 | -2.243 | -0.855 | 4.654 | 3.597 | 4.968 | 3.051 |
| CaCIPK13 | -0.416 | -0.716 | 0.996 | 0.320 | -0.739 | -1.337 | -0.445 | -0.251 | -2.449 | -2.659 | -1.244 | -1.698 | -0.167 | -3.610 | -0.054 | -0.704 | 1.391 | -1.517 | | -1.425 | -1.232 | -1.325 | 0.000 | -1.814 | -1.056 | -1.167 | 3.210 | -2.099 |
| CaCIPK14 | 5.413 | 5.324 | 4.661 | 5.514 | 5.938 | 6.673 | 4.796 | 5.683 | 4.133 | 5.803 | 6.506 | 4.154 | 5.783 | 5.244 | 5.374 | 6.350 | 5.396 | 4.589 | | 5.093 | 5.206 | 3.457 | 3.172 | 3.627 | 5.603 | 5.906 | 5.449 | 4.166 |
| CaCIPK15 | 7.382 | 6.870 | 5.273 | 5.529 | 4.491 | 4.562 | 4.436 | 5.931 | 3.869 | 6.520 | 5.885 | 4.737 | 5.327 | 5.967 | 3.613 | 4.501 | 2.938 | 4.007 | | 6.307 | 6.306 | 1.031 | 2.172 | 1.437 | 4.752 | 6.273 | 4.608 | 3.958 |
| CaCIPK16 | 5.289 | 4.346 | 4.780 | 5.416 | 5.144 | 4.500 | 5.139 | 3.562 | 4.569 | 5.182 | 4.287 | 3.795 | 5.407 | 5.836 | 4.084 | 5.719 | 6.278 | 5.257 | | 5.803 | 5.845 | 3.336 | -0.243 | 3.519 | 6.175 | 5.487 | 6.619 | 4.760 |
| CaCIPK17 | 0.832 | 0.864 | -1.258 | 1.670 | 0.458 | 2.579 | 2.053 | 1.149 | 1.411 | 1.758 | 3.129 | 0.000 | 2.207 | 0.753 | 0.955 | 0.425 | -1.657 | 1.751 | | 2.593 | 2.859 | -3.925 | -2.926 | -3.351 | 1.842 | 1.506 | 2.342 | -1.153 |
| CaCIPK18 | 4.530 | 4.333 | 4.410 | 5.983 | 4.135 | 5.339 | 4.101 | 5.169 | 4.734 | 5.991 | 5.375 | 4.704 | 5.418 | 5.887 | 5.219 | 6.185 | 5.403 | 6.119 | | 4.643 | 4.706 | 2.470 | 1.072 | 2.721 | 5.149 | 5.234 | 4.311 | 4.649 |
| CaCIPK19 | 0.000 | -0.465 | 0.000 | 0.000 | 0.000 | 0.000 | 0.000 | 0.000 | 0.000 | 0.000 | 0.000 | 0.000 | 0.000 | -1.733 | 0.000 | -0.490 | 0.000 | 0.000 | | 0.000 | 0.000 | -2.036 | 0.992 | 0.000 | 0.000 | 0.000 | 0.000 | 0.000 |
| CaCIPK20 | -3.837 | 0.000 | 0.000 | 0.000 | 0.000 | 0.000 | -5.020 | -5.280 | 0.000 | 0.000 | 0.000 | 0.000 | -4.056 | 8.005 | 1.921 | -3.367 | -2.552 | -1.044 | | -5.223 | -4.399 | 0.000 | 0.000 | -4.313 | 0.000 | 0.000 | -1.773 | 0.000 |
| CaCIPK21 | 0.000 | -1.251 | 0.000 | -9.715 | 0.000 | 0.000 | -3.111 | -1.751 | -1.289 | -1.537 | -2.977 | 0.000 | 0.000 | -0.274 | -5.108 | 0.000 | -0.852 | -3.550 | | -2.263 | 0.000 | -1.476 | 0.000 | -2.271 | -7.441 | 0.000 | -2.465 | -5.051 |
| CaCIPK22 | 2.839 | 2.961 | 3.032 | 5.030 | 3.207 | 4.748 | 5.426 | 4.591 | 4.765 | 5.717 | 5.567 | 3.912 | 4.373 | 5.461 | 4.919 | 4.950 | 4.802 | 5.179 | | 5.659 | 5.549 | 0.159 | 1.438 | 0.130 | 5.140 | 5.325 | 3.857 | 4.665 |

**Table S8:** Log2 Fold Change values of CaCIPKs in various stages of seed development.

|  | **SEED STAGE 1** | | **SEED STAGE 2** | | **SEED STAGE 3** | | **SEED STAGE 4** | | **SEED STAGE 5** | | **SEED STAGE 6** | | **SEED STAGE 7** | |
| --- | --- | --- | --- | --- | --- | --- | --- | --- | --- | --- | --- | --- | --- | --- |
| **GENE** | **JGK3** | **HIMCHANA1** | **JGK3** | **HIMCHANA1** | **JGK3** | **HIMCHANA1** | **JGK3** | **HIMCHANA1** | **JGK3** | **HIMCHANA1** | **JGK3** | **HIMCHANA1** | **JGK3** | **HIMCHANA1** |
| CaCIPK1 | -2.648 | -1.964 | -0.522 | -0.097 | -0.817 | 0.398 | -0.086 | 0.464 | -0.213 | -1.114 | -3.006 | -2.411 | -2.977 | -2.135 |
| CaCIPK2 | 2.360 | 2.453 | 1.789 | 1.847 | 1.637 | 1.372 | 2.677 | 3.548 | 3.234 | 1.404 | 5.204 | 3.481 | 5.040 | 1.961 |
| CaCIPK3 | -0.625 | -0.196 | -0.051 | 0.329 | -0.289 | -1.064 | -1.041 | -0.555 | -0.163 | -0.496 | -0.924 | -0.660 | -0.225 | -0.257 |
| CaCIPK4 | 0.101 | 0.961 | 0.743 | 1.292 | -0.785 | 1.087 | 0.085 | 1.810 | -1.383 | -0.479 | -2.655 | -0.403 | -2.410 | -0.036 |
| CaCIPK5 | -1.347 | -1.082 | -0.855 | -0.633 | -1.069 | -0.397 | -0.271 | -0.786 | -2.796 | -3.618 | -0.502 | 0.094 | -0.542 | 1.113 |
| CaCIPK6 | -0.515 | -0.297 | 0.768 | 0.809 | -0.732 | -0.208 | -0.605 | -0.246 | -2.152 | -3.680 | -3.514 | -3.354 | -3.779 | -3.545 |
| CaCIPK7 | -0.281 | -0.860 | 0.467 | -0.265 | -1.211 | -1.376 | -0.475 | -0.716 | -1.827 | -0.864 | -1.171 | -0.778 | -2.002 | -0.297 |
| CaCIPK9 | -0.274 | -0.433 | -0.127 | -0.698 | -0.546 | -2.460 | -0.794 | -0.550 | -0.502 | -4.233 | -4.151 | -2.852 | -2.044 | -2.064 |
| CaCIPK10 | -2.533 | -2.511 | -1.263 | -1.931 | -2.458 | -2.577 | -0.035 | -0.033 | 0.516 | 0.154 | -0.610 | -2.098 | -1.306 | -2.892 |
| CaCIPK11 | 6.421 | 5.647 | 7.195 | 6.910 | 5.883 | 6.104 | 6.508 | 6.238 | 4.177 | 4.020 | 4.010 | 2.766 | 5.752 | 2.005 |
| CaCIPK12 | -3.112 | -3.016 | -2.559 | -2.069 | -2.309 | -2.183 | -2.347 | -2.564 | -2.915 | -4.507 | -3.905 | -4.191 | -3.134 | -4.191 |
| CaCIPK13 | 5.183 | 4.290 | 5.909 | 4.723 | 5.497 | 4.100 | 4.719 | 3.202 | 4.819 | 3.722 | 0.131 | -0.446 | 0.000 | -1.257 |
| CaCIPK14 | 0.671 | 0.601 | 1.410 | 1.169 | 0.543 | 0.853 | 0.720 | 0.696 | -0.702 | -0.963 | -1.623 | -0.202 | -0.947 | 0.421 |
| CaCIPK15 | -0.962 | -1.278 | -0.466 | -0.094 | -1.383 | -2.016 | -0.839 | -1.005 | -2.734 | -1.112 | -1.081 | -1.078 | -1.682 | 0.124 |
| CaCIPK16 | -1.114 | -0.284 | 0.340 | 0.804 | -0.677 | -0.127 | -0.888 | -0.099 | -2.347 | -3.791 | -4.804 | -4.249 | -5.235 | -4.463 |
| CaCIPK17 | -6.550 | -5.754 | -4.590 | -3.573 | -7.096 | -5.911 | -5.892 | -6.034 | -5.460 | -6.153 | -6.638 | -7.327 | -6.587 | -8.327 |
| CaCIPK18 | 1.553 | 1.106 | 2.255 | 1.330 | 1.223 | 1.353 | 2.119 | 2.093 | 1.086 | -0.174 | -1.920 | -2.629 | -1.749 | -2.466 |
| CaCIPK21 | 0.000 | 3.769 | 0.000 | 3.007 | 0.000 | 3.470 | 0.000 | 1.018 | 0.000 | 2.108 | 0.000 | 0.000 | 0.000 | -1.327 |
| CaCIPK22 | -1.973 | -1.904 | -1.989 | -1.329 | -3.666 | -2.125 | -2.863 | -2.272 | -6.082 | -5.465 | -6.213 | -5.952 | -6.288 | -6.648 |

**Table S9:** qPCR expression data of CaCIPKs under drought, salt and ABA treatments in chickpea.

| **Root** | | | | | | | | | | |
| --- | --- | --- | --- | --- | --- | --- | --- | --- | --- | --- |
| **Drought** | | | | | | | | | | |
|  |  | **1hr** | | | **3hr** | | | **6hr** | | |
| **Gene** | **Gene ID** | Fold Change | Regulation | P value | Fold Change | Regulation | P value | Fold Change | Regulation | P value |
| ***CaCIPK2*** | **LOC101488582** | 3.3 | UP | 0.04 | 2.05 | UP | 0.23 | 6.59 | UP | 0.0002 |
| ***CaCIPK11*** | **LOC101499591** | 4.3 | UP | 0.03 | 7.87 | UP | 0.02 | 1.48 | UP | 0.3 |
| ***CaCIPK12*** | **LOC101506657** | 20.6 | UP | 0.003 | 2.9 | UP | 0.04 | 71 | UP | 0.01 |
| ***CaCIPK16*** | **LOC101510050** | 0.2 | DOWN | 0.03 | 5.18 | UP | 0.02 | 0.07 | DOWN | 0.01 |
| ***CaCIPK17*** | **LOC101510187** | 7.64 | UP | 0.14 | 5.18 | UP | 0.018 | 7.64 | UP | 0.0009 |
| ***CaCIPK21*** | **LOC101512343** | 6.9 | UP | 0.007 | 1.53 | UP | 0.025 | 2.2 | UP | 0.24 |
|  |  |  |  |  |  |  |  |  |  |  |
| **Shoot** | | | | | | | | | | |
| **Drought** | | | | | | | | | | |
|  |  | **1hr** | | | **3hr** | | | **6hr** | | |
| **Gene** | **Gene ID** | Fold Change | Regulation | P value | Fold Change | Regulation | P value | Fold Change | Regulation | P value |
| ***CaCIPK2*** | **LOC101488582** | 4.17 | UP | 0.03 | 18 | UP | 0.001 | 9.33 | UP | 0.003 |
| ***CaCIPK11*** | **LOC101499591** | 2.3 | UP | 0.0009 | 6 | UP | 0.002 | 5.3 | UP | 0.02 |
| ***CaCIPK12*** | **LOC101506657** | 0.2 | DOWN | 0.03 | 15 | UP | 0.004 | 3.07 | UP | 0.1 |
| ***CaCIPK16*** | **LOC101510050** | 0.84 | DOWN | 0.59 | 3.2 | UP | 0.04 | 5.4 | UP | 0.04 |
| ***CaCIPK17*** | **LOC101510187** | 0.56 | DOWN | 0.31 | 1.3 | UP | 0.03 | 1.5 | UP | 0.23 |
| ***CaCIPK21*** | **LOC101512343** | 1.5 | UP | 0.21 | 1.13 | UP | 0.23 | 0.92 | DOWN | 0.11 |

**Figure S1:** Percentage of identity and similarity between *CaCIPKs.*


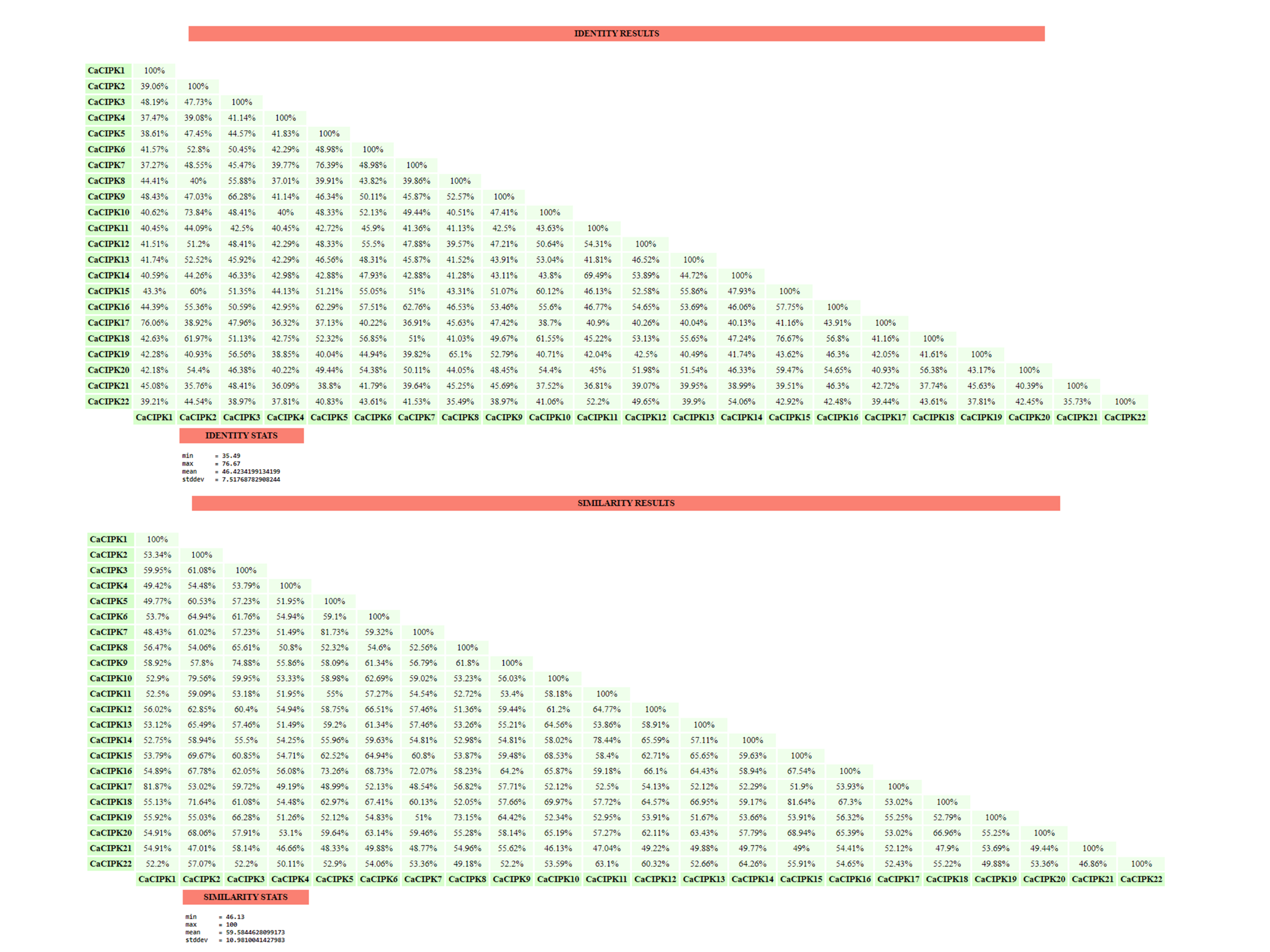


**Figure S2:** Alignment of 22 CaCIPKs for domain identification.

**
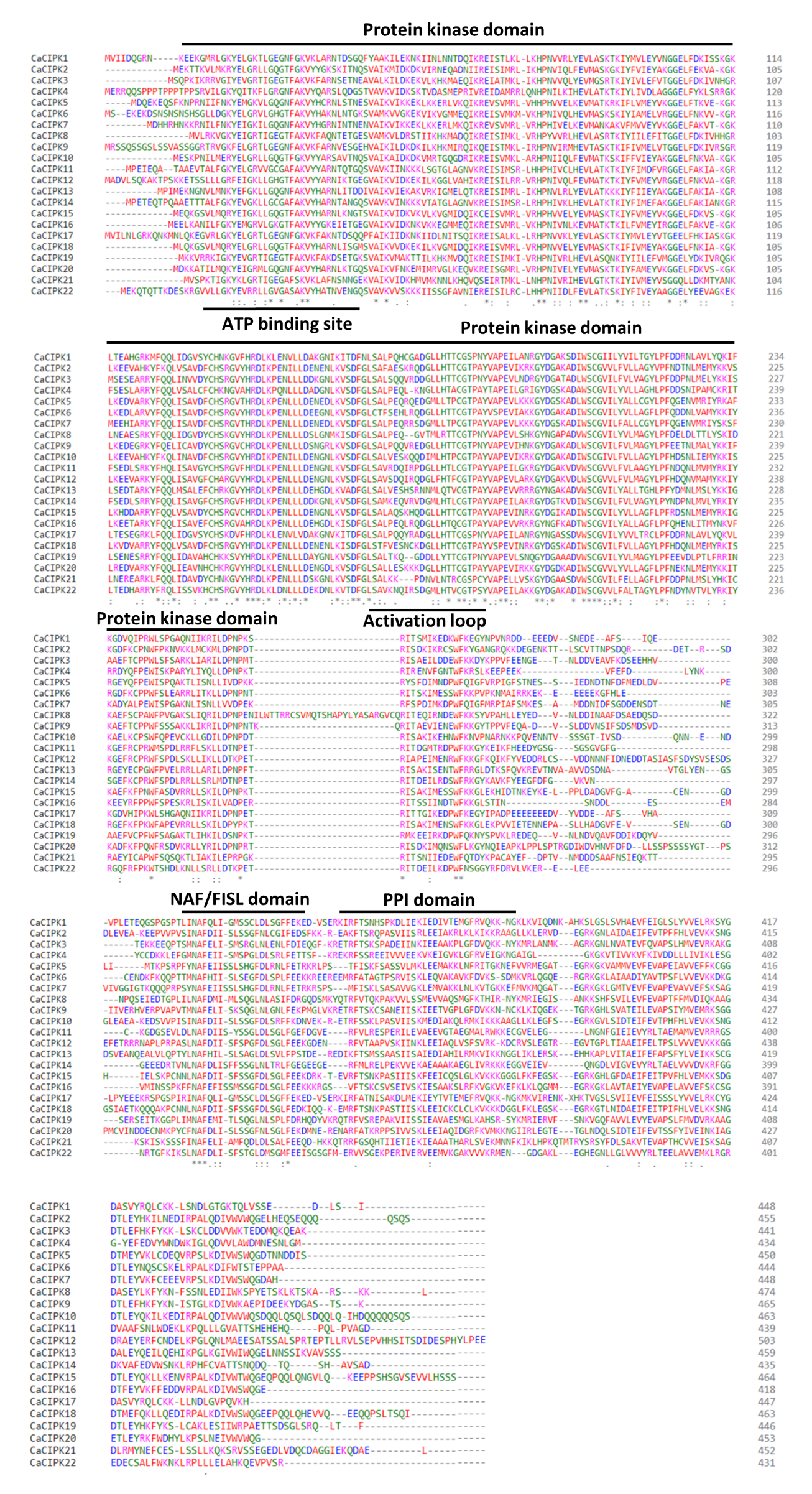
**

**Figure S3:** Identification of motifs through MEME.

**
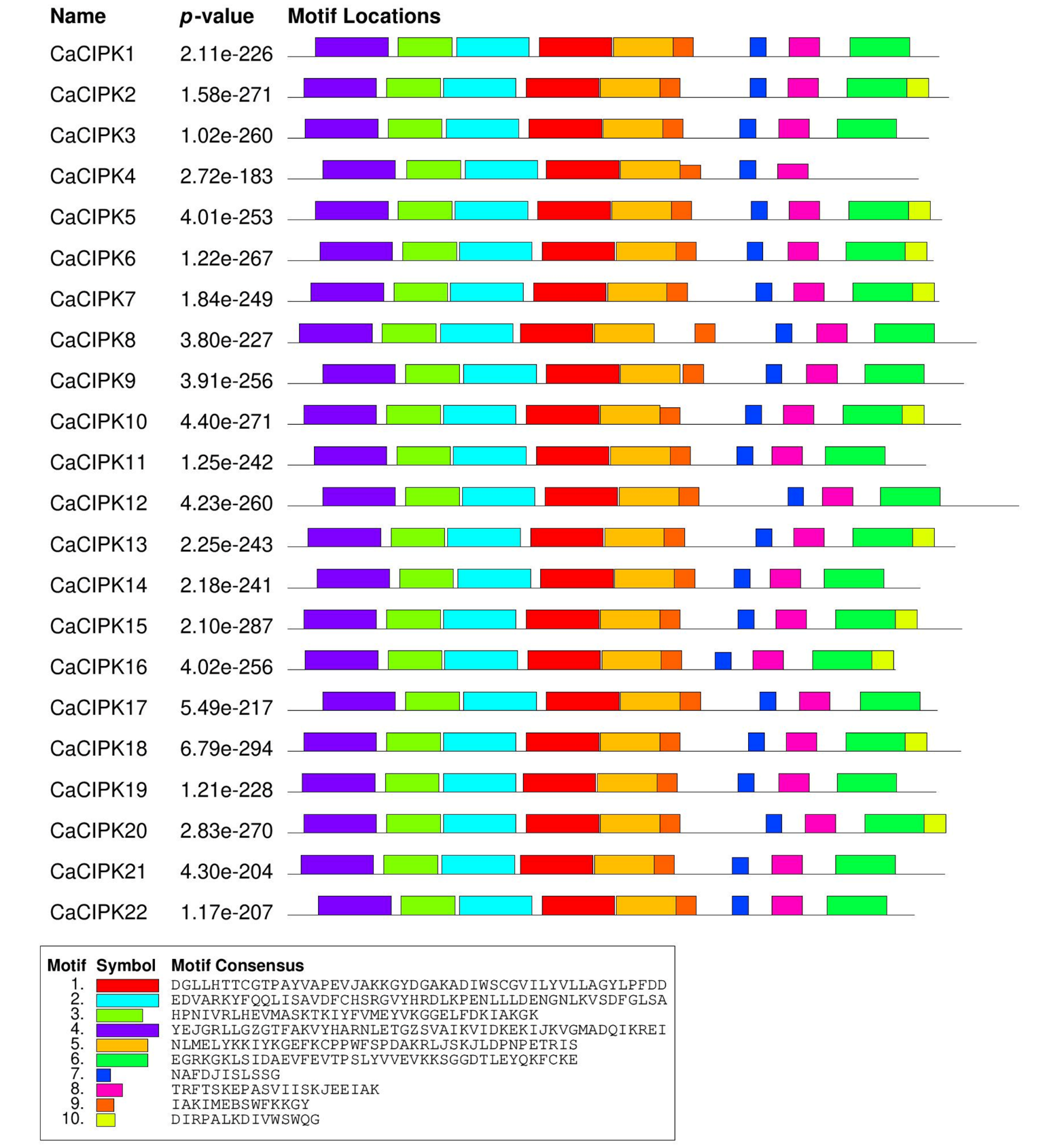
**

**Figure S4:** Duplication of chickpea *CIPK* genes performed by MCScanX is shown via Circos plot.

**
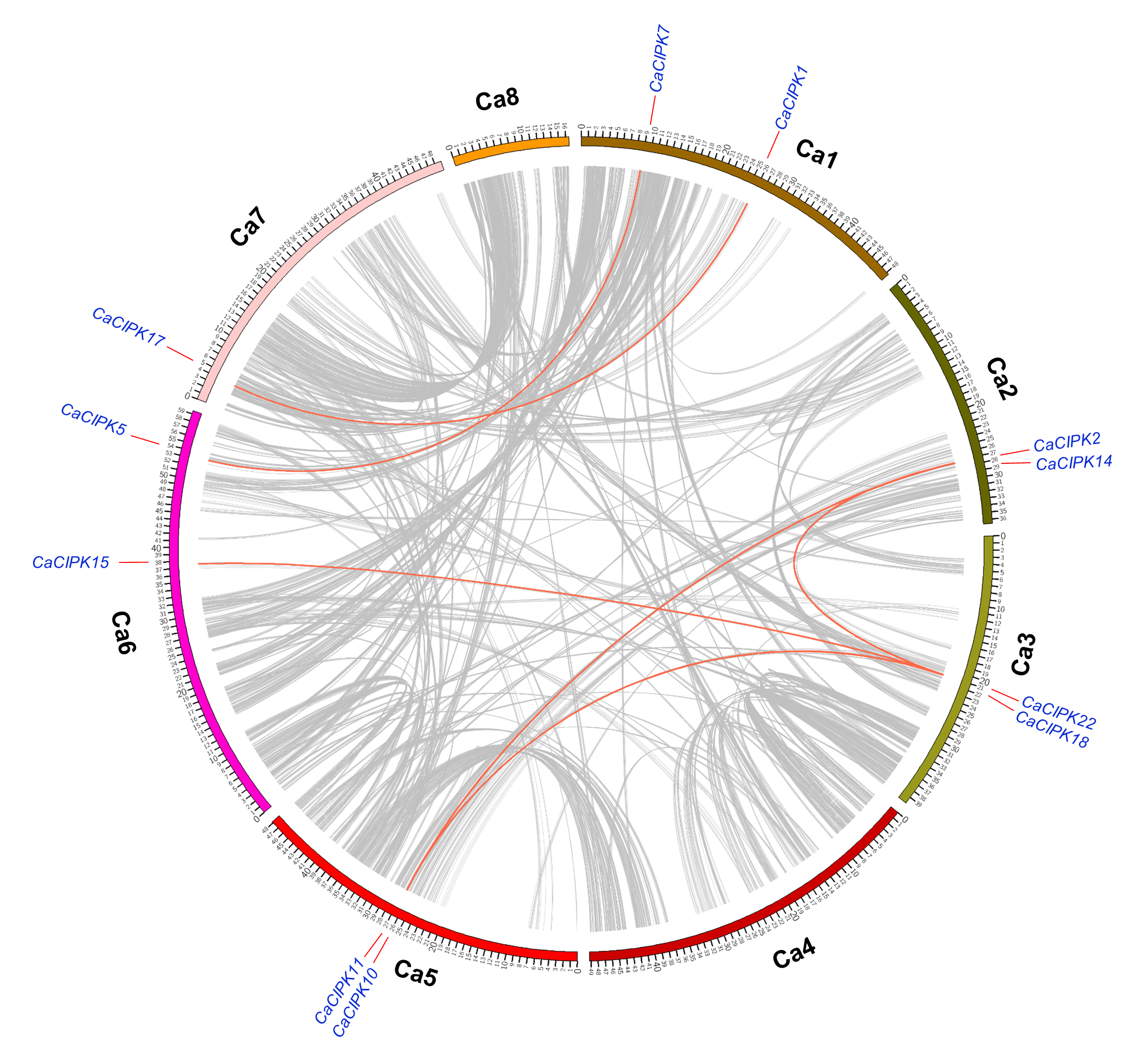
**

**Figure S5.** Expression profiles of different *CIPK* genes in different developmental stages of pigeonpea.

**
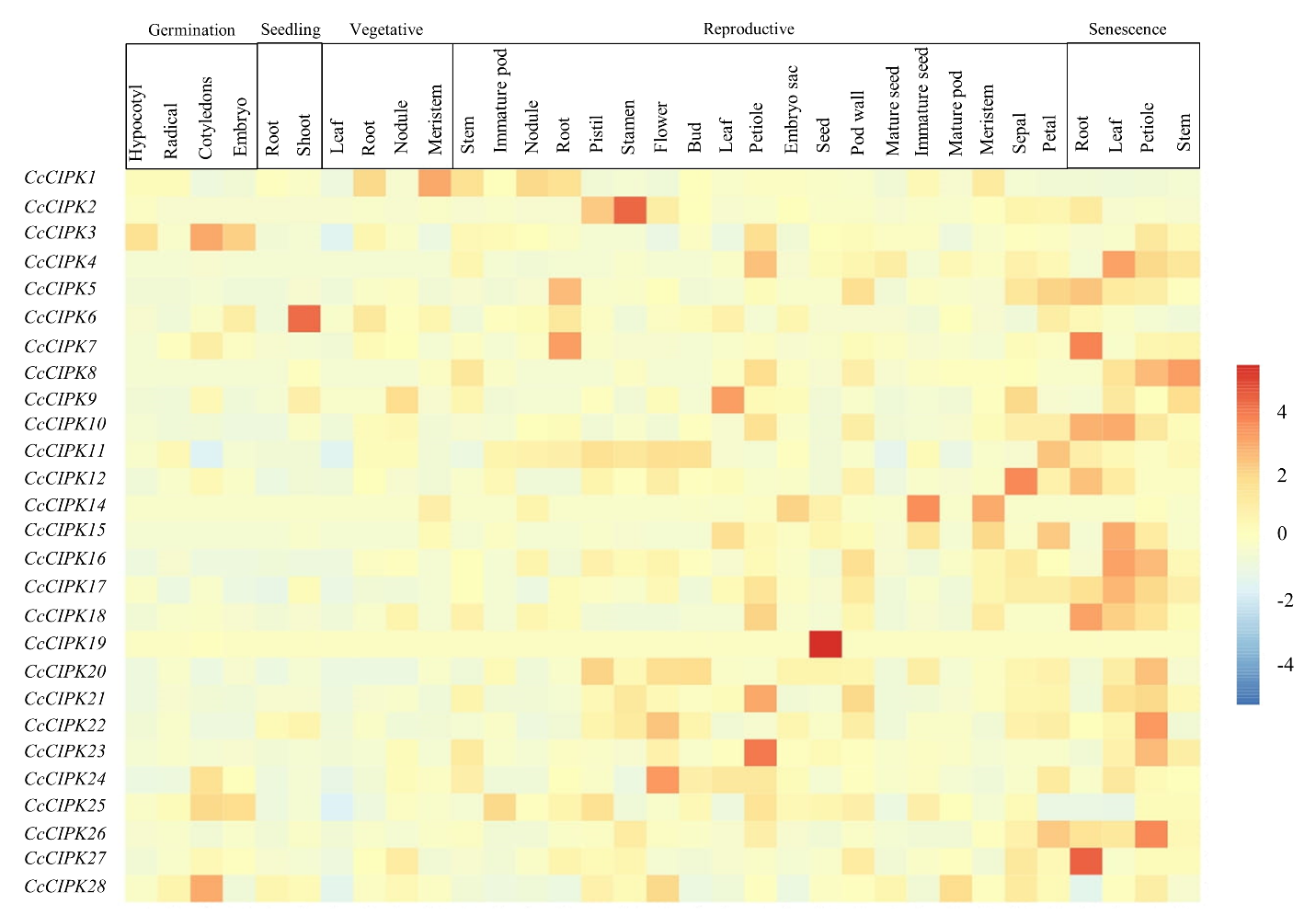
**

**Figure S6.** Expression profiles of *CIPK* genes under heat and salt stress in pigeonpea. The terms SS and ST in the figure correspond to “Salt Susceptible” and “Salt Tolerant” respectively.

**
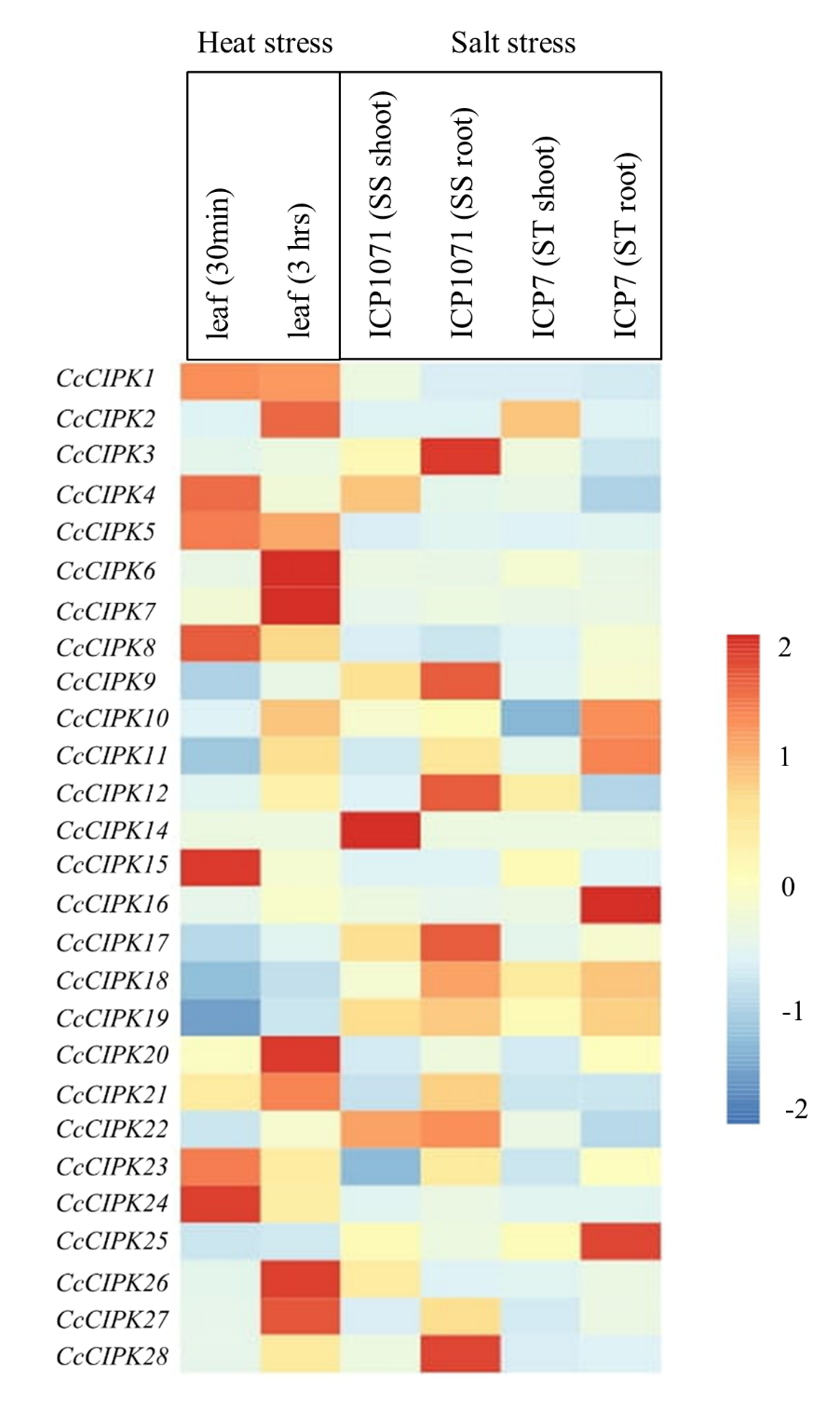
**
